# Supplementary material for: Structure of giant kelp Photosystem I-FCP uncovers drivers of antenna evolution across the red lineage
Source: Nat Commun. 2026 May 21;17:4772. doi: 10.1038/s41467-026-73499-x (PMC13219633; doi:10.1038/s41467-026-73499-x)
Supplement: Supplementary file 1 — Supplementary Information [file 41467_2026_73499_MOESM1_ESM.pdf]

Supplementary Information

Structure of giant kelp Photosystem I-FCP uncovers drivers of antenna evolution across the red lineage

Jenevieve D. Weissman[1], Pablo Maturana[1], Hui M. O. Oung[1], Reece Riddle[1,2], Gabrielle Wyatt[1], Viktoria G. T. Dubinin[1], Philipp Zerbe[1], María Maldonado[1,3*]

Supplementary Items:

Supplementary Figures 1 to 16

Supplementary Tables 1 to 5

Supplementary Notes 1 to 3

Supplementary Methods

Supplementary References

Supplementary Figures

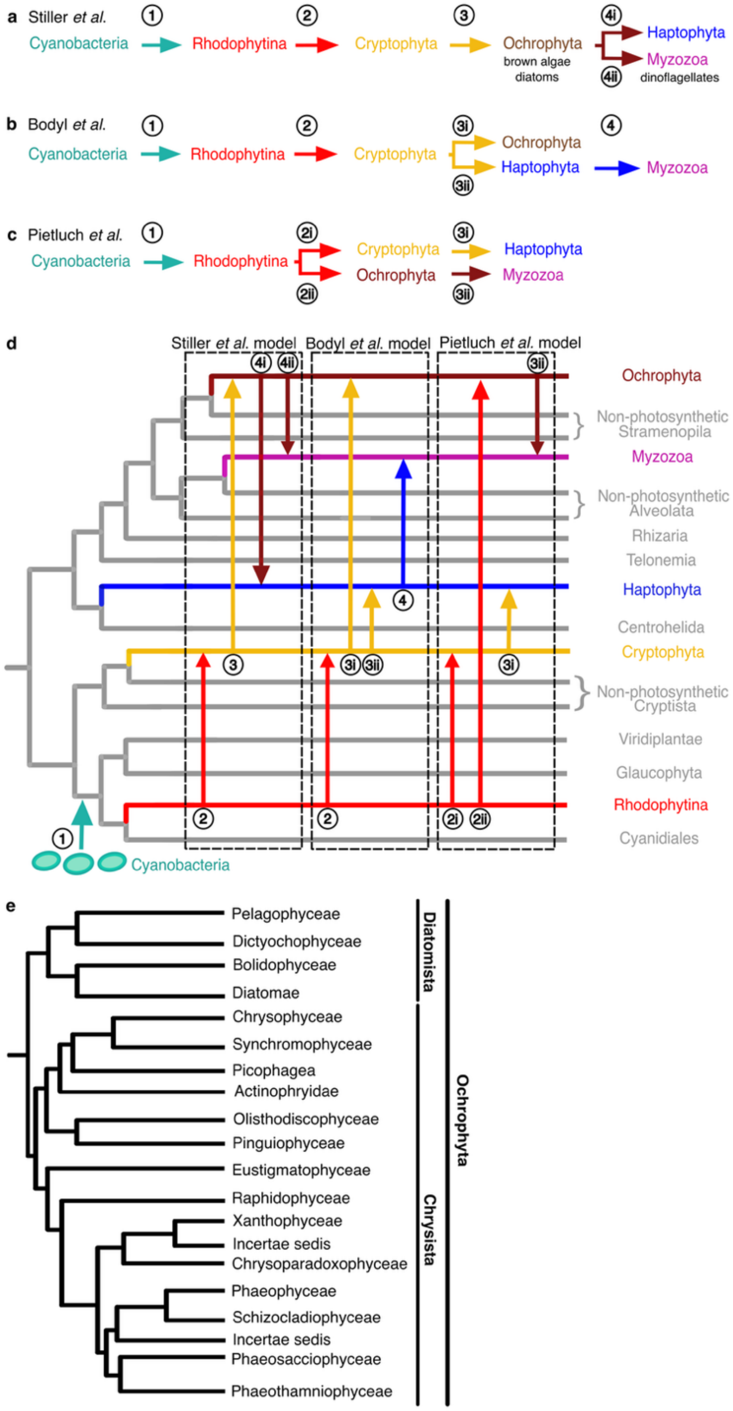

Supplementary Fig. 1. Evolutionary relationships in the red lineage. a-c Models of endosymbioses in the red lineage consistent with molecular timescale analysis[25,26] proposed by Stiller *et al.*[27] (a) Bodyl *et al.*[28] (b) and Pietluch *et al.*[26] (c). Arrows and numbers represent serial and parallel endosymbioses. Numbers indicate level of serial endosymbiosis (e.g., primary, secondary). Endosymbioses marked with (i) or (ii) indicate two separate endosymbiosis events at the same level. **d** Representation of (a-c) on simplified cladogram. Colours and labelling as in (a-c). **e** Cladogram of Ochrophyta phylum[33].

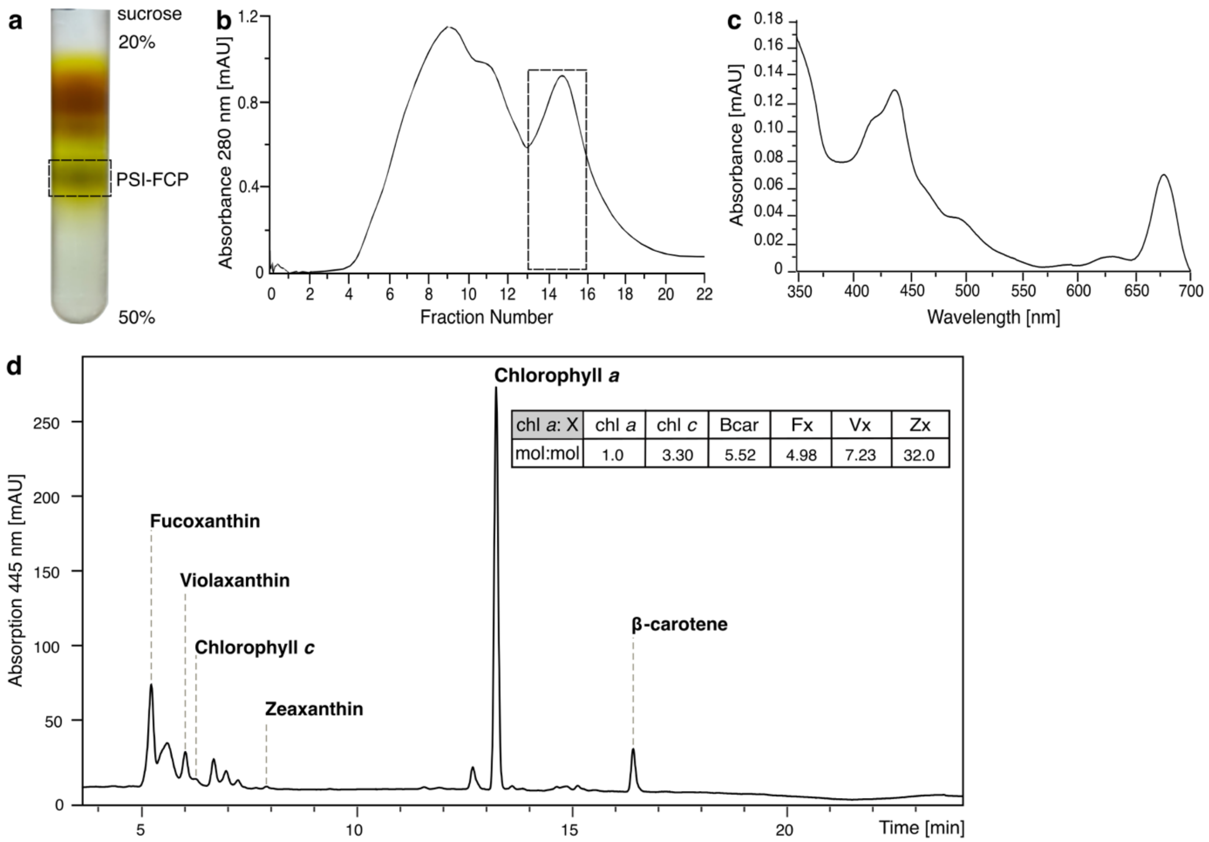

Supplementary Fig. 2. Purification and biochemical characterization of *Macrocystis pyrifera*'s PSI-FCP supercomplex. a Linear sucrose gradient (20-50%, w:v) before fractionation. **b** Chromatogram of the fractions from (a), showing the absorbance monitored at 280 nm. Fractions that were selected and pooled for subsequent cryoEM processing are indicated in a dashed box. **c** Absorption spectrum monitored at 350-750 nm of the pooled purified fractions indicated in (b). **d** Chromophore analysis of purified fractions using high-performance liquid chromatography (HPLC) based on absorption at 445 nm. Chromophores were identified by comparison to authentic standards. Inset table shows ratios (mole:mole) of chl *a* relative to the other chromophores. chl *a*, chlorophyll *a*; chl *c*, chlorophyll *c*; BCar, ß-carotene; Fx, fucoxanthin; Vx, violaxanthin; Zx, zeaxanthin.

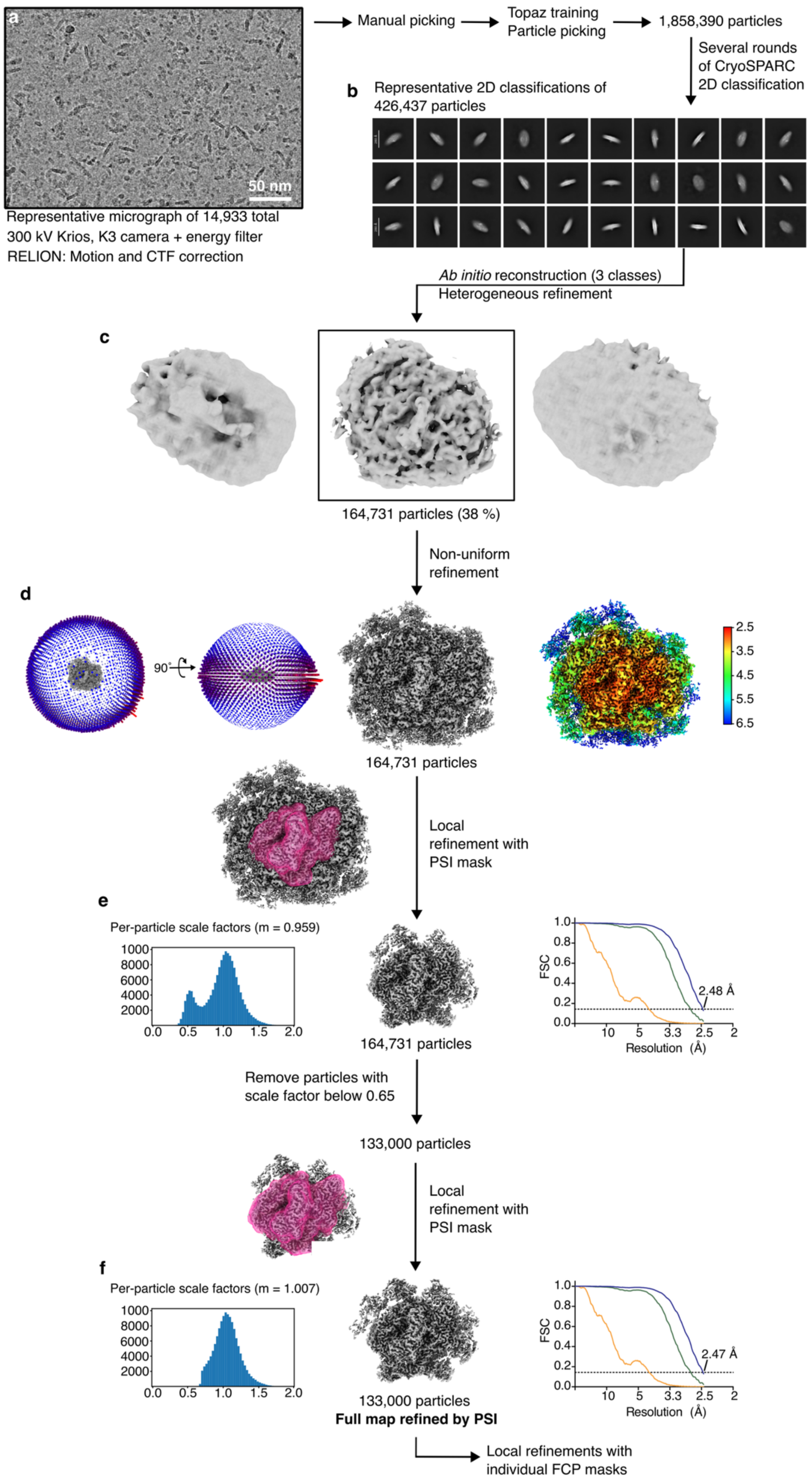
a
50 nm
Representative micrograph of 14,933 total
300 kV Krios, K3 camera + energy filter
RELION: Motion and CTF correction
Manual picking
Topaz training
Particle picking
1,858,390 particles
Several rounds
of CryoSPARC
2D classification
b
Representative 2D classifications of
426,437 particles
Ab initio reconstruction (3 classes)
Heterogeneous refinement
c
164,731 particles (38 %)
Non-uniform
refinement
d
90°
2.5
3.5
4.5
5.5
6.5
164,731 particles
Local
refinement with
PSI mask
e
Per-particle scale factors (m = 0.959)
1000
8000
6000
4000
2000
0.0
0.5
1.0
1.5
2.0
164,731 particles
FSC
1.0
0.8
0.6
0.4
0.2
0.0
2.48 Å
10
5
3.3
2.5
2
Resolution (Å)
Remove particles with
scale factor below 0.65
133,000 particles
Local
refinement with
PSI mask
f
Per-particle scale factors (m = 1.007)
1000
8000
6000
4000
2000
0.0
0.5
1.0
1.5
2.0
133,000 particles
Full map refined by PSI
FSC
1.0
0.8
0.6
0.4
0.2
0.0
2.47 Å
10
5
3.3
2.5
2
Resolution (Å)
Local refinements with
individual FCP masks

Supplementary Fig. 3. (previous page) **CryoEM micrograph and initial data processing for *Macrocystis pyrifera*'s PSI-FCP supercomplex. a** Representative micrograph of 14,933 collected. **b** Representative 2D classes of PSI-FCP particles. **c-e** Initial 3D classification and refinement pipeline to obtain the full map refined by PSI (**f**). **c** *Ab initio* reconstruction and heterogeneous refinement, with selection of best-resolved class. **d** Non-uniform refinement. Left to right: viewing direction distribution, density map and local resolution of initial map with full particle set (2.5-6.5 Å). Note that local resolutions of FPCs significantly improve after further classification and masked local refinements (Supplementary Figs. 4-6). **e** Local refinement with PSI mask (magenta) with full particle set. Left to right: per-particle scale factor distribution, density map, Fourier shell correlation (FSC) curves shown for no (orange), loose (green) and tight (blue) masking. Resolution at which tight masked FSC crosses 0.143 gold-standard limit (dashed line) is shown. **f** Local refinement with PSI mask with downscaled particles below 0.65 removed. From left to right as (**e**). Note that the final map refined by PSI (**f**) was used as the starting point for the local refinement of individual FCPs shown in Supplementary Fig. 4-6.

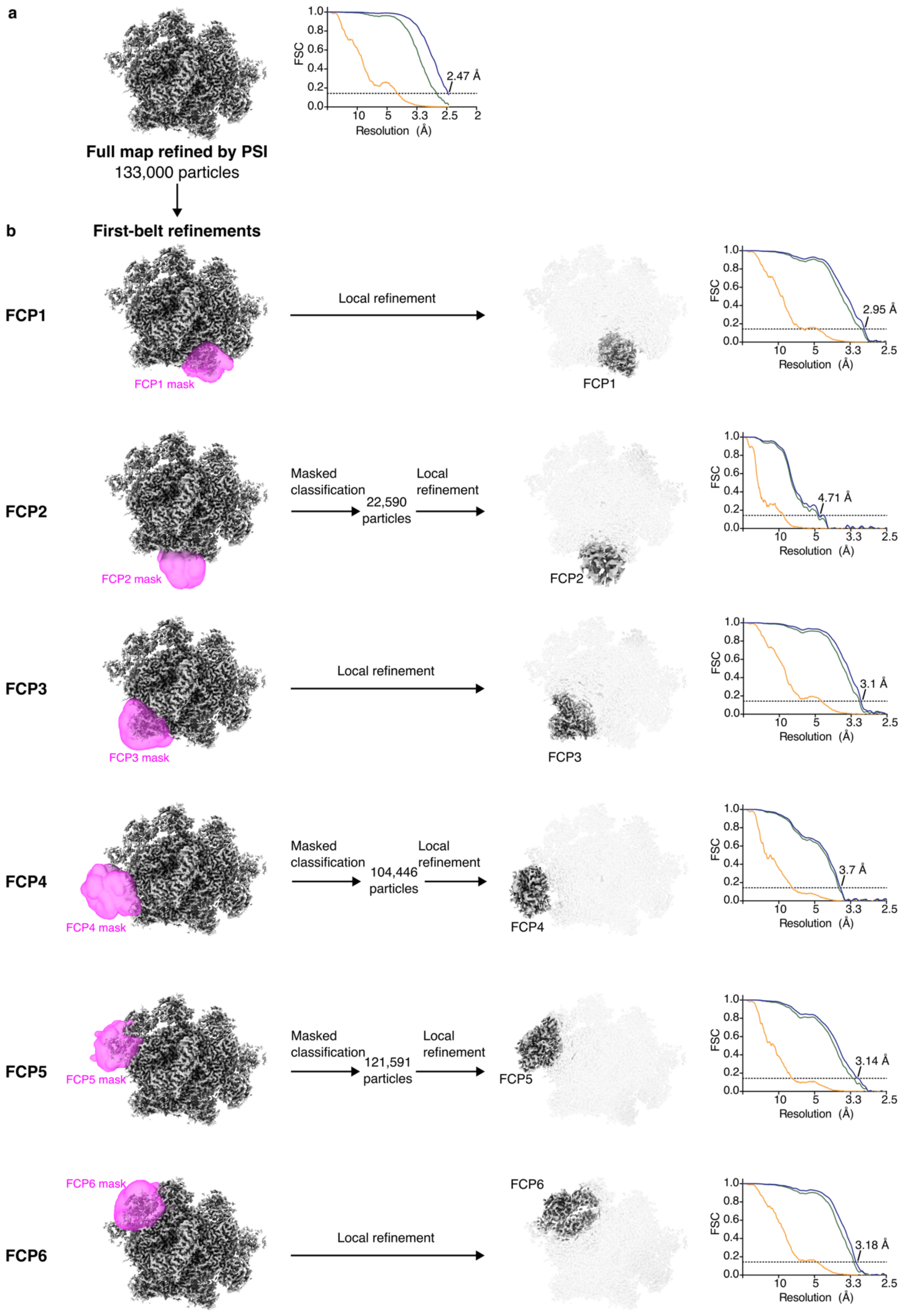
a
Full map refined by PSI
133,000 particles
2.47 Å
FSC
Resolution (Å)
b
First-belt refinements
FCP1
FCP1 mask
Local refinement
2.95 Å
FCP2
FCP2 mask
Masked classification
22,590 particles
Local refinement
4.71 Å
FCP3
FCP3 mask
Local refinement
3.1 Å
FCP4
FCP4 mask
Masked classification
104,446 particles
Local refinement
3.7 Å
FCP5
FCP5 mask
Masked classification
121,591 particles
Local refinement
3.14 Å
FCP6
FCP6 mask
Local refinement
3.18 Å

Supplementary Fig. 4. (previous page) **Masked classification and focused refinements of FCP1-FCP6 (first belt). a** Starting map for the focused refinements was map refined by PSI and corresponding particle stack (Supplementary Fig. 3f). **b** Focused refinements using individual masks (magenta) for FCP1-6. Due to sub-stoichiometric occupancy of the FCP2/4/5/6, masked classifications using corresponding individual mask was performed before focused refinement of those specific regions, using the same mask. Fourier shell correlation (FSC) curves for individual focused refinements are shown for no (orange), loose (green) and tight (blue) masking. Resolution at which the tight mask FSC crosses 0.143 gold-standard limit (dashed line) is shown.

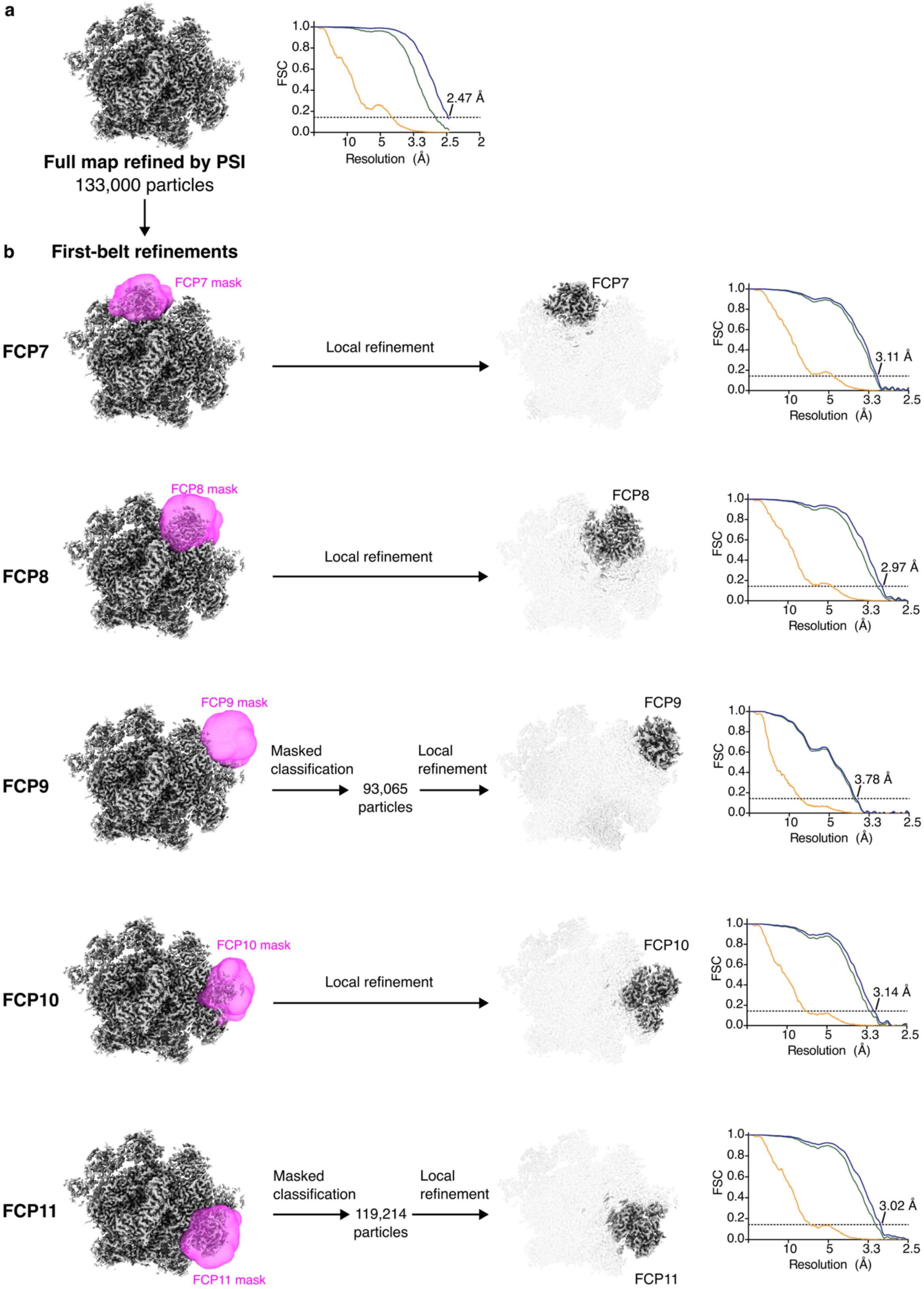

a
Full map refined by PSI
133,000 particles
FSC
Resolution (Å)
2.47 Å
b
First-belt refinements
FCP7
FCP7 mask
Local refinement
3.11 Å
FCP8
FCP8 mask
Local refinement
2.97 Å
FCP9
FCP9 mask
Masked classification
93,065 particles
Local refinement
3.78 Å
FCP10
FCP10 mask
Local refinement
3.14 Å
FCP11
FCP11 mask
Masked classification
119,214 particles
Local refinement
3.02 Å

Supplementary Fig. 5. (previous page) **Masked classification and focused refinements of FCP7-FCP11 (first belt). a** Starting map for the focused refinements was map refined by PSI and its corresponding particle stack (Supplementary Fig. 3f). **b** Focused refinements using individual masks (magenta) for FCP7-11. For FCP9/11, masked classifications using corresponding individual mask was performed before focused refinement of those specific regions, using the same mask. Fourier shell correlation (FSC) curves for individual focused refinements are shown for no (orange), loose (green) and tight (blue) masking. Resolution at which the tight mask FSC crosses 0.143 gold-standard limit (dashed line) is shown.

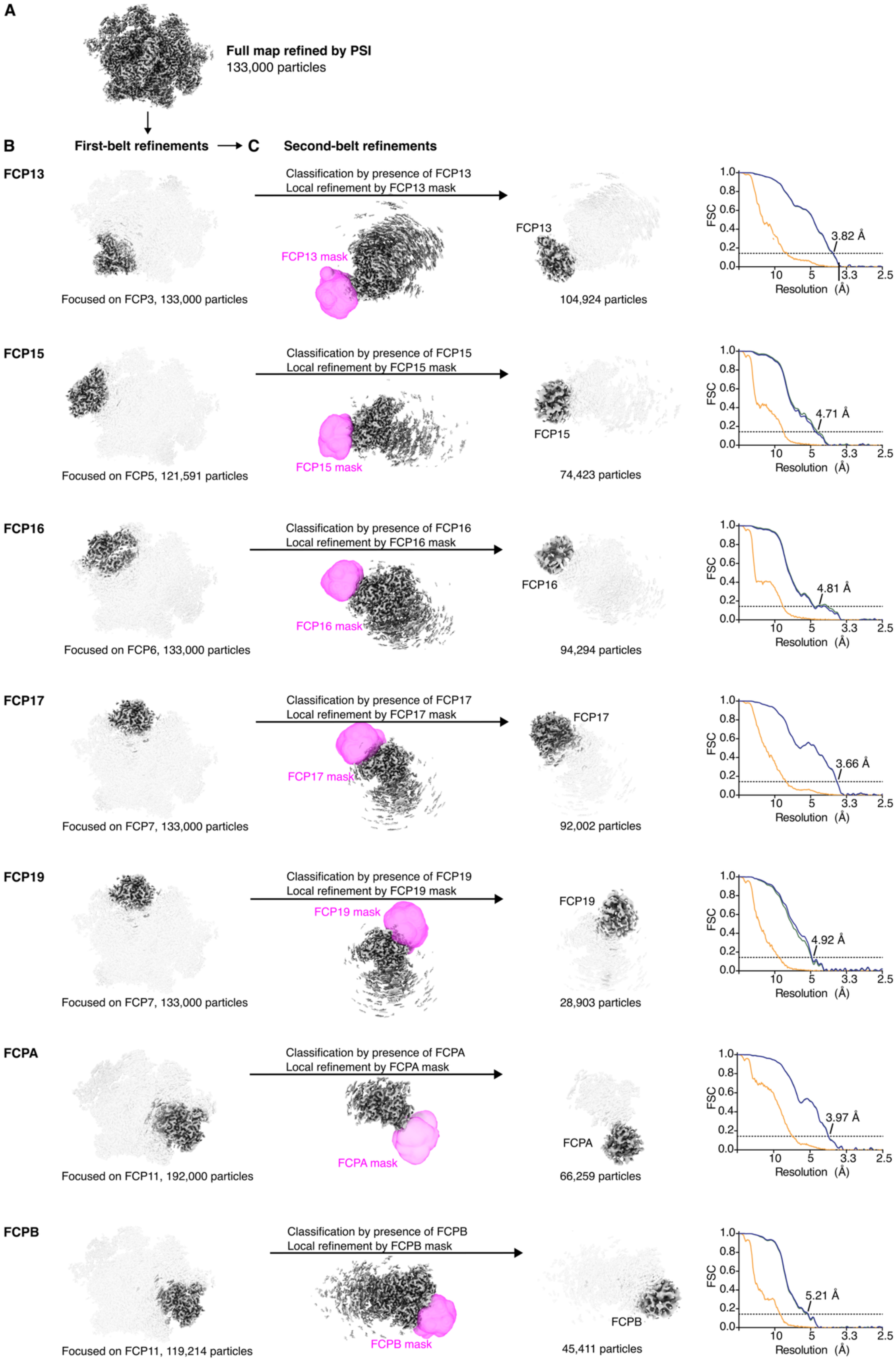
A
Full map refined by PSI
133,000 particles
B
First-belt refinements
C
Second-belt refinements
FCP13
Classification by presence of FCP13
Local refinement by FCP13 mask
FCP13 mask
FCP13
Focused on FCP3, 133,000 particles
104,924 particles
3.82 Å
FCP15
Classification by presence of FCP15
Local refinement by FCP15 mask
FCP15 mask
FCP15
Focused on FCP5, 121,591 particles
74,423 particles
4.71 Å
FCP16
Classification by presence of FCP16
Local refinement by FCP16 mask
FCP16 mask
FCP16
Focused on FCP6, 133,000 particles
94,294 particles
4.81 Å
FCP17
Classification by presence of FCP17
Local refinement by FCP17 mask
FCP17 mask
FCP17
Focused on FCP7, 133,000 particles
92,002 particles
3.66 Å
FCP19
Classification by presence of FCP19
Local refinement by FCP19 mask
FCP19 mask
FCP19
Focused on FCP7, 133,000 particles
28,903 particles
4.92 Å
FCPA
Classification by presence of FCPA
Local refinement by FCPA mask
FCPA mask
FCPA
Focused on FCP11, 192,000 particles
66,259 particles
3.97 Å
FCPB
Classification by presence of FCPB
Local refinement by FCPB mask
FCPB mask
FCPB
Focused on FCP11, 119,214 particles
45,411 particles
5.21 Å
FSC
Resolution (Å)

Supplementary Fig. 6. (previous page) **Masked classification and focused refinements of FCP13/15/16/17/19/A/B (second-belt). a-c** Focused refinements performed in three subsequent steps: (a) refinement by PSI (Supplementary Fig. 3f), (b) refinement by relevant first-belt FCP (Supplementary Fig. 4-5), (c) masked classification and refinement by second-belt FCP, using masks shown (magenta). Note that particles for FCPA were obtained from expanded initial PSI particle set (192,000 particles). Fourier shell correlation (FSC) curves for individual focused refinements are shown for no (orange), loose (green) and tight (blue) masking. Resolution at which the tight mask FSC crosses 0.143 gold-standard limit (dashed line) is shown.

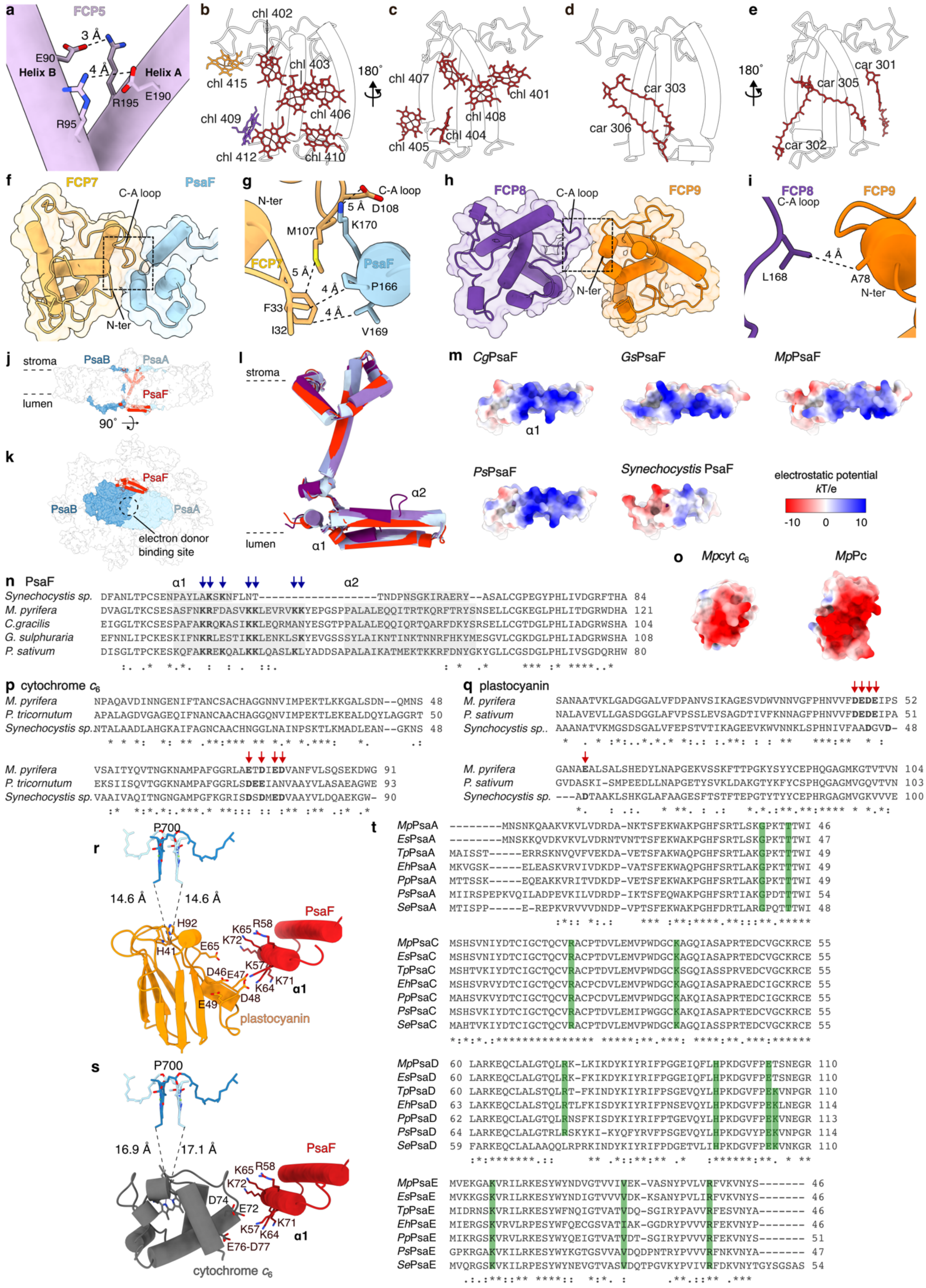

a
FCP5
3 Å
E90
4 Å
Helix B
Helix A
E190
R195
R95
b
chl 402
chl 403
chl 415
chl 406
chl 409
chl 412
chl 410
180˚
c
chl 407
chl 401
chl 408
chl 405
chl 404
d
car 303
car 306
e
180˚
car 301
car 305
car 302
f
FCP7
PsaF
C-A loop
N-ter
g
C-A loop
N-ter
5 Å
D108
K170
M107
FCP7
PsaF
5 Å
4 Å
P166
F33
4 Å
I32
V169
h
FCP8
C-A loop
FCP9
N-ter
i
FCP8
C-A loop
FCP9
4 Å
L168
A78
N-ter
j
stroma
PsaB
PsaA
PsaF
lumen
90˚
k
PsaF
PsaB
PsaA
electron donor
binding site
l
stroma
α2
lumen
α1
m
CgPsaF
GsPsaF
MpPsaF
α1
PsPsaF
Synechocystis PsaF
electrostatic potential
kT/e
-10
0
10
o
Mpcyt c6
MpPc
n PsaF
α1
α2
Synechocystis sp. DFANLTPCSENPAYLAKSKNFLNT------------------TNDPNSGKIRAERY--ASALCGPEGYPHLIVDGRFTHA 84
M. pyrifera DVAGLTKCSESASFNKRFDASVKKLEVRVKKYEPGSPPALALEQQITRTKQRFTRYSNSELLCGKEGLPHLITDGRWDHA 121
C.gracilis EIGGLTKCSESPAFAKRQKASIKKLEQRMANYESGTPPALALEQQIQRTQARFDKYSRSELLCGTDGLPHLIADGRWSHA 104
G. sulphuraria EFNNLIPCKESKIFNKRLESTIKKLENKLSKYEVGSSSYLAIKNTINKTNNRFHKYMESGVLCGKDGLPHLIADGRWSHA 108
P. sativum DISGLTPCKESKQFAKREKQALKKLQASLKLYADDSAPALAIKATMEKTKKRFDNYGKYGLLCGSDGLPHLIVSGDQRHW 80
p cytochrome c6
M. pyrifera NPAQAVDINNGENIFTANCSACHAGGNNVIMPEKTLKKGALSDN--QMNS 48
P. tricornutum APALAGDVGAGEQIFNANCAACHAGGQNVIMPEKTLEKEALDQYLAGGRT 50
Synechocystis sp. NTALAADLAHGKAIFAGNCAACHNGGLNAINPSKTLKMADLEAN--GKNS 48
M. pyrifera VSAITYQVTNGKNAMPAFGGRLAETDIEDVANFVLSQSEKDWG 91
P. tricornutum EKSIISQVTGGKNAMPAFGGRLSDEEIANVAAYVLASAEAGWE 93
Synechocystis sp. VAAIVAQITNGNGAMPGFKGRISDSDMEDVAAYVLDQAEKGW- 90
q plastocyanin
M. pyrifera SANAATVKLGADGGALVFDPANVSIKAGESVDWVNNVGFPHNVVFDEDEIPS 52
P. sativum NALAVEVLLGASDGGLAFVPSSLEVSAGDTIVFKNNAGFPHNVVFDEDEIPA 51
Synchocystis sp.. AAANATVKMGSDSGALVFEPSTVTIKAGEEVKWVNNKLSPHNIVFAADGVD- 48
M. pyrifera GANAEALSALSHEDYLNAPGEKVSSKFTTPGKYSYYCEPHQGAGMKGTVTVN 104
P. sativum GVDASKI-SMPEEDLLNAPGETYSVKLDAKGTYKFYCSPHQGAGMVGQVTVN 103
Synechocystis sp. ---ADTAAKLSHKGLAFAAGESFTSTFTEPGTYTYYCEPHRGAGMVGKVVVE 100
r
P700
14.6 Å
14.6 Å
H92
PsaF
K65
R58
H41
K72
E65
K57
D46
E47
K71
K64
α1
E49
D48
plastocyanin
s
P700
16.9 Å
17.1 Å
PsaF
K65
R58
K72
D74
E72
K71
K57
K64
α1
E76-D77
cytochrome c6
t
MpPsaA --------MNSNKQAAKVKVLVDRDA-NKTSFEKWAKPGHFSRTLSKGPKTTTWI 46
EsPsaA --------MNSKKQVDKVKVLVDRNTVNTTSFEKWAKPGHFSRTLSKGPKTTTWI 47
TpPsaA MAISST-----ERRSKNVQVFVEKDA-VETSFAKWAQPGHFSRTLAKGPKTTTWI 49
EhPsaA MKVGSK-----ELEASKVRVIVDKDP-VATSFEKWAQPGHFSRTLAKGPKTTTWI 49
PpPsaA MTTSSK-----EQEAKKVRITVDKDP-VETSFEKWAKPGHFSRTLAKGPKTTTWI 49
PsPsaA MIIRSPEPKVQILADPEVKILVDRDP-IKTSFEQWAKPGHFSRTIAKGPDTTTWI 54
SePsaA MTISPP-----E-REPKVRVVVDNDP-VPTSFEKWAKPGHFDRTLARGPQTTTWI 48
MpPsaC MSHSVNIYDTCIGCTQCVRACPTDVLEMVPWDGCKAGQIASAPRTEDCVGCKRCE 55
EsPsaC MSHSVNIYDTCIGCTQCVRACPTDVLEMVPWDGCKAGQIASAPRTEDCVGCKRCE 55
TpPsaC MSHTVKIYDTCIGCTQCVRACPTDVLEMVPWDGCKSGQIASSPRVEDCVGCKRCE 55
EhPsaC MSHSVRVYDTCIGCTQCVRACPCDVLEMVPWDGCKAGQIASAPRAEDCIGCKRCE 55
PpPsaC MAHSVKVYDTCIGCTQCVRACPCDVLEMVPWDGCKAKQIASAPRTEDCIGCKRCE 55
PsPsaC MSHSVKIYDTCIGCTQCVRACPTDVLEMIPWGGCKAKQIASAPRTEDCVGCKRCE 55
SePsaC MAHTVKIYDTCIGCTQCVRACPTDVLEMVPWDGCKAGQIASSPRTEDCVGCKRCE 55
MpPsaD 60 LARKEQCLALGTQLRK-LKIKDYKIYRIFPGGEIQFLHPKDGVFPETSNEGR 110
EsPsaD 60 LARKEQCLALGTQLRK-FKIKDYKIYRIFPGGEIQFLHPKDGVFPETSNEGR 110
TpPsaD 60 LARKEQCLALSTQLRT-FKINDYKIYRIFPSGEVQYLHPKDGVFPEKVNPGR 110
EhPsaD 63 LAKKEQCLALSTQLRTLFKISDYKIYRIFPNSEVQYLHPKDGVFPEKLNEGR 114
PpPsaD 62 LARKEQCLALGTQLRNSFKISDYKIYRIFPTGEVQYLHPKDGVFPEKVNEGR 113
PsPsaD 64 LARKEQCLALGTRLRSKYKI-KYQFYRVFPSGEVQYLHPKDGVYPEKVNPGR 114
SePsaD 59 FARKEQCLALAAQQLRPRKINDYKIYRIFPDGETVLIHPKDGVFPEKVNKGR 110
MpPsaE MVEKGAKVRILRKESYWYNDVGTVVIVEK-VASNYPVLVRFVKVNYS------- 46
EsPsaE MVKKGSKVRILRKESYWYNDIGTVVVVDK-KSANYPVLIRFVKVNYS------- 46
TpPsaE MIDRNSKVRILRKESYWFNQIGTVATVDQ-SGIRYPAVVRFESVNYA------- 46
EhPsaE MIERGSKVRVLRPESYWFQECGSVATIAK-GGDRYPVVVRFEKVNYP------- 46
PpPsaE MIKRGSKVRVLRKESYWYQEIGTVATVDT-SGIRYPVVVRFEKVNYS------- 51
PsPsaE GPKRGAKVKILRKESYWYKGTGSVVAVDQDPNTRYPVVVRFNKVNYA------- 47
SePsaE MVQRGSKVKILRPESYWYNEVGTVASVDQTPGVKYPVIVRFDKVNYTGYSGSAS 54

Supplementary Fig. 7. (previous page) **Further structural details for *M. pyrifera* PSI-FCP. a** Example of FCP arg-glu salt bridges between helix A/B. **b-e** *M. pyrifera* FCP-chromophore binding sites shown for chlorophyll (b, c) and carotenoids (d, e) in core-facing orientation (b, d) or outer-belt-facing orientation (c, e). Family-specific chromophores shown in subfamily colour (Lhcr-orange, RedCAP-red), non-family-specific shown in brown. **f-g** Example of first-belt FCP interacting with PSI subunit through FCP C-A and N-terminal (N-ter) loops, viewed from stroma. Subunits shown in cartoon with semi-transparent surface overlay. Area in dashed box in (f) is detailed in (g). **h-i** Example of two first-belt FCPs interacting through the A-C and N-terminal loops, viewed from stroma. Subunits shown in cartoon with semi-transparent surface overlay, coloured by subunit. Dashed box in (h) detailed in (i). **j**, **k** Membrane (j) and lumenal (k) views of *M. pyrifera* PSI-FCP in surface representation. PsaA/B/F coloured by subunit; PsaA/B in surface; PsaF in cartoon. Approximate location of electron-donor binding site marked in dashed circle. **l** Structural superposition of PsaF from *Chaetoceros gracilis* (light blue, PDB: 6L4U), *Galdieria sulphuraria* (red alga, dark blue, PDB:9KC5), *M. pyrifera* (red, PDB: 9YGV), *Pisum sativum* (light violet, PDB: 4XK8), and *Synechocystis sp.* (dark violet, PDB:5OY0). **m** Electrostatic surface of PsaF-α1 helix from *G. gracilis*, *G. sulphuraria gracilis*, *M. pyrifera*, *P. sativum*, and *Synechocystis sp*. Red, negative; white, neutral; blue, positive. **n** Sequence alignment of the N-terminal region of PsaF from *Synechocystis sp*., *M. pyrifera*, *C. gracilis*. *G. sulphuraria*, *P. sativum*, with α1–α2 helices (grey boxes) and conserved basic residues (blue arrows). **o** Electrostatic surfaces of predicted models of *M. pyrifera* (Mp) cytochrome c_6 (cyt c_6) and plastocyanin (Pc). Scale as in (m). **p, q** Sequence alignments of cyt c_6 from *M. pyrifera*, *P. tricornutum*, and *Synechocystis sp*. PCC 680 (p) and Pc from *M. pyrifera*, *P.sativum*, and *Synechocystis sp*. PCC 6803 (q). Conserved acidic residues at docking interface marked with red arrows. **r, s** Docking models of *M. pyrifera* Pc (r) and cyt c_6 (s) bound to *M. pyrifera* PSI. **t** Sequence alignments of regions of PsaA, PsaC, PsaD and PsaE implicated in ferredoxin binding from *M. pyrifera* (brown alga, *Mp*), *Ectocarpus siliculosus* (brown alga, *Es*), *Thalassiosira pseudonana* (diatom, *Tp*), *Emiliania huxleyi* (haptophyte, *Eh*), *Porphyridium purpurea* (red alga, *Pp*), *P. sativum* (plant, *Ps*), and *S. elongatus* (cyanobacterium, *Se*). Conserved residues associated with ferredoxin binding marked in green boxes.

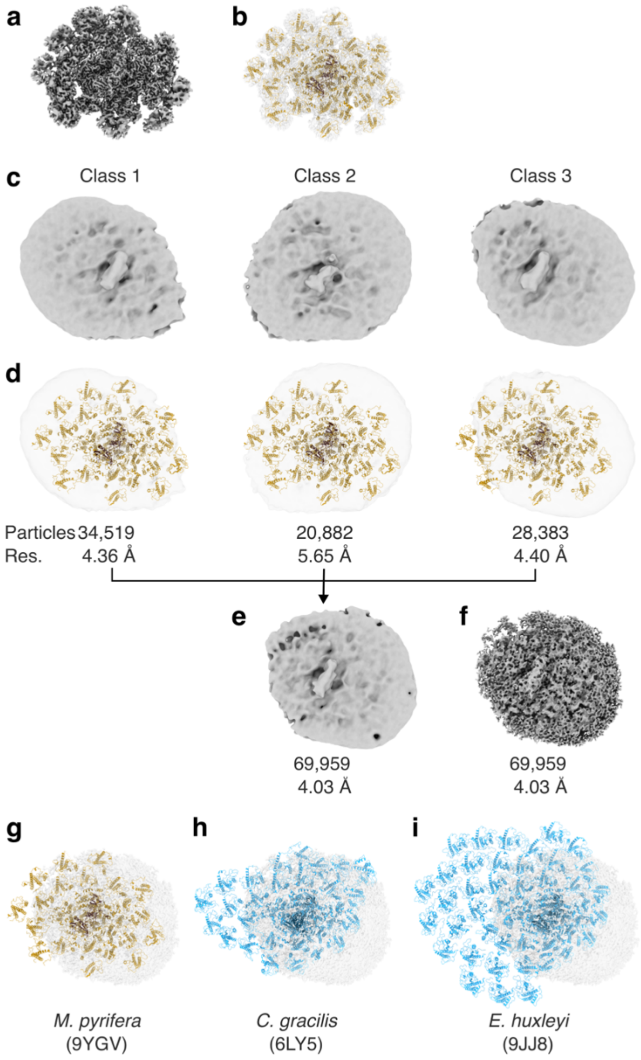

Supplementary Fig. 8. Larger PSI-FCP antenna from *M. pyrifera*. a, b Map (a) and model superposed on semitransparent map (b) of *M. pyrifera* PSI-FCP supercomplex discussed in all other figures or this work (PDB: 9YGV). Model shown in cartoon, coloured in brown. Stromal domain (PsaC/D/E) coloured in darker brown as visual anchor. **c-f** Larger PSI-FCP supercomplex obtained from *M. pyrifera* preparation extracted in different conditions relative to (a,b). **c, d** Reconstructions of three PSI-FCP supercomplex classes (class 1-3)obtained after non-uniform refinement. To reduce high-resolution noise and make the antenna architecture more evident, maps were Gaussian-filtered with 3 standard deviations. Maps shown alone (c) or with the *M. pyrifera* model (PDB: 9YGV, this work) fitted and superposed. Coloured as in (b). Particle numbers and resolutions (Res.) for each class are indicated. **e, f** Reconstruction of the combined particles from classes 1-3 in (c,d) obtained after non-uniform refinement. Reconstruction shown with (e) or without (f) Gaussian filter as in (c,d). **g, i** Reconstruction from (f) fitted with superposed models of PSI-FCP supercomplexes from *M. pyrifera* (PDB: 9YGV, this work) (g), diatom *C. gracilis* (PDB: 6LY5)[1] (h) or *E. huxleyi* (PDB: 9JJ8)[14] (i). Models shown in cartoon and coloured in brown with stromal domain in darker brown (g) or in blue with stromal domain in darker blue (h, i).

a

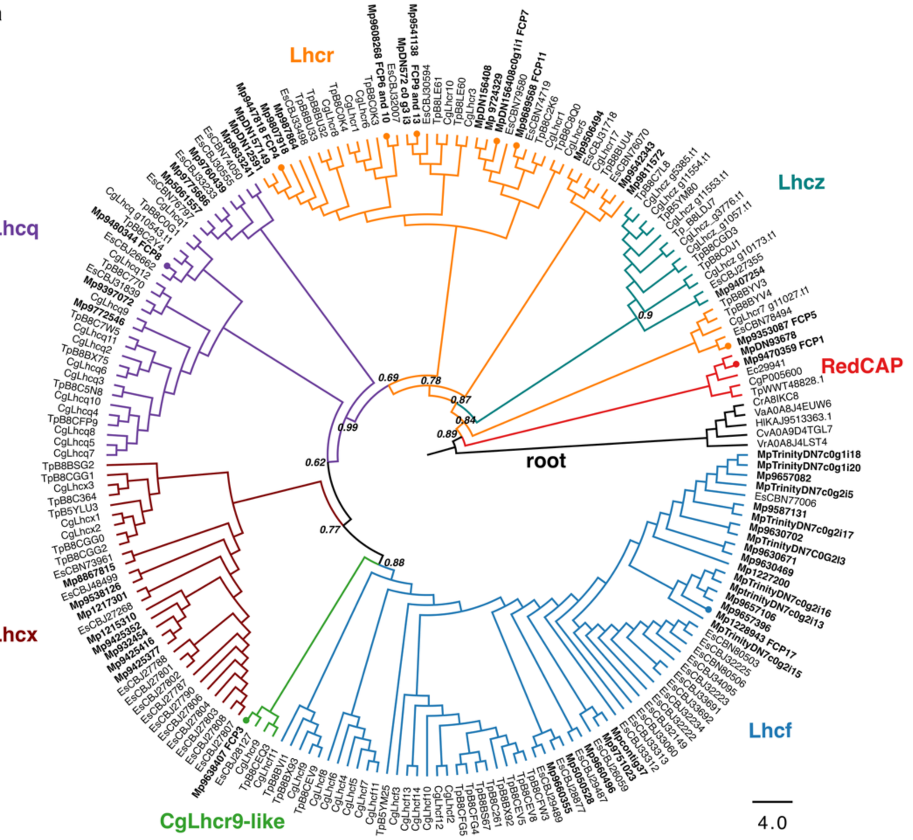
Lhcr
Lhcz
Lhcq
RedCAP
root
Lhcx
Lhcf
CgLhcr9-like
4.0
0.69
0.78
0.87
0.84
0.89
0.99
0.62
0.77
0.88
0.9

b

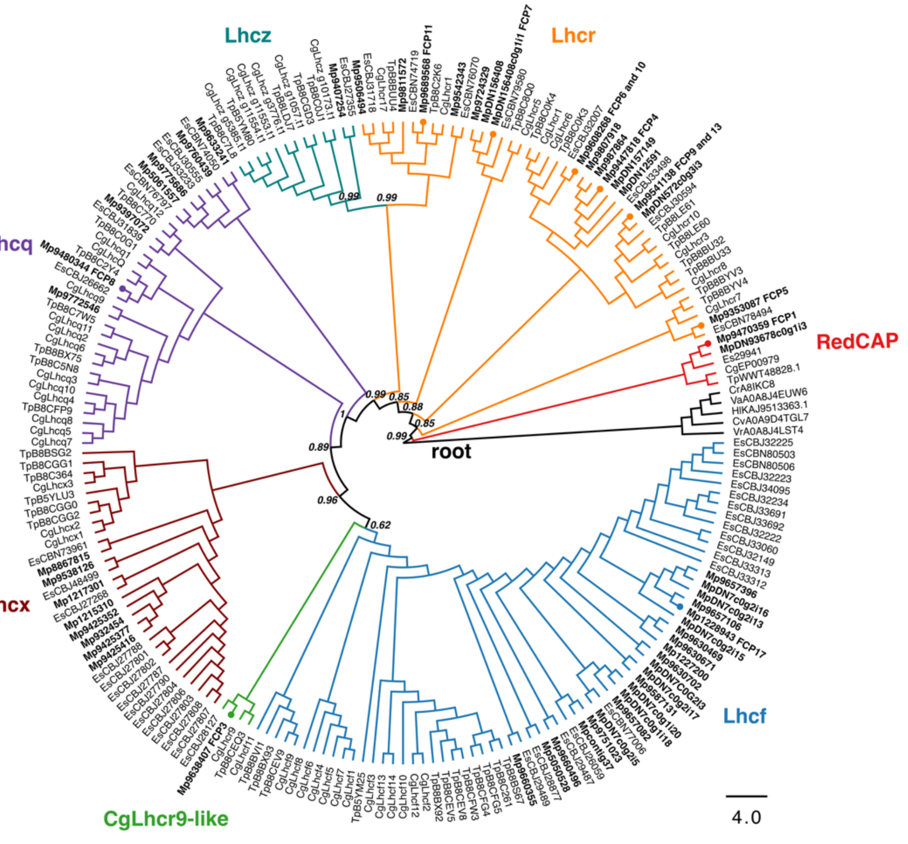
Lhcz
Lhcr
Lhcq
RedCAP
root
Lhcx
Lhcf
CgLhcr9-like
4.0
0.99
0.99
0.99
0.85
0.88
0.85
0.99
0.89
0.96
0.62

Supplementary Fig. 9. (previous page) **Phylogenetic analysis of ochrophyte FCP proteins. a, b** Maximum likelihood phylogenetic tree with branch support values calculated using SH-aLRT test (a) or aBayes (b). Both trees were inferred from 203 LHC protein sequences from *Macrocystis pyrifera* (Mp), *Ectocarpus siliculosus* (Es), *Chaetoceros gracilis* (Cg), and *Thalassiosira pseudonana* (Tp). FCP subfamilies indicated by colour: Lhcr (orange), Lhcf (blue), Lhcq (purple), Lhcx (brown), Lhcz (teal) and CgLhcr9-like (green); RedCAP of LHC superfamily in red. Black branches correspond to green algal LHC sequences used to root the trees. *M. pyrifera* sequences are highlighted in bold; those identified in the *M. pyrifera* PSI-FCP structure (PDB: 9YGV, this work) indicated with circle at branch terminus. Posterior probabilities supporting basal nodes that define each subfamily shown beside the node. Scale indicates number of substitutions per site. Sequence details are provided in Supplementary Data 1.

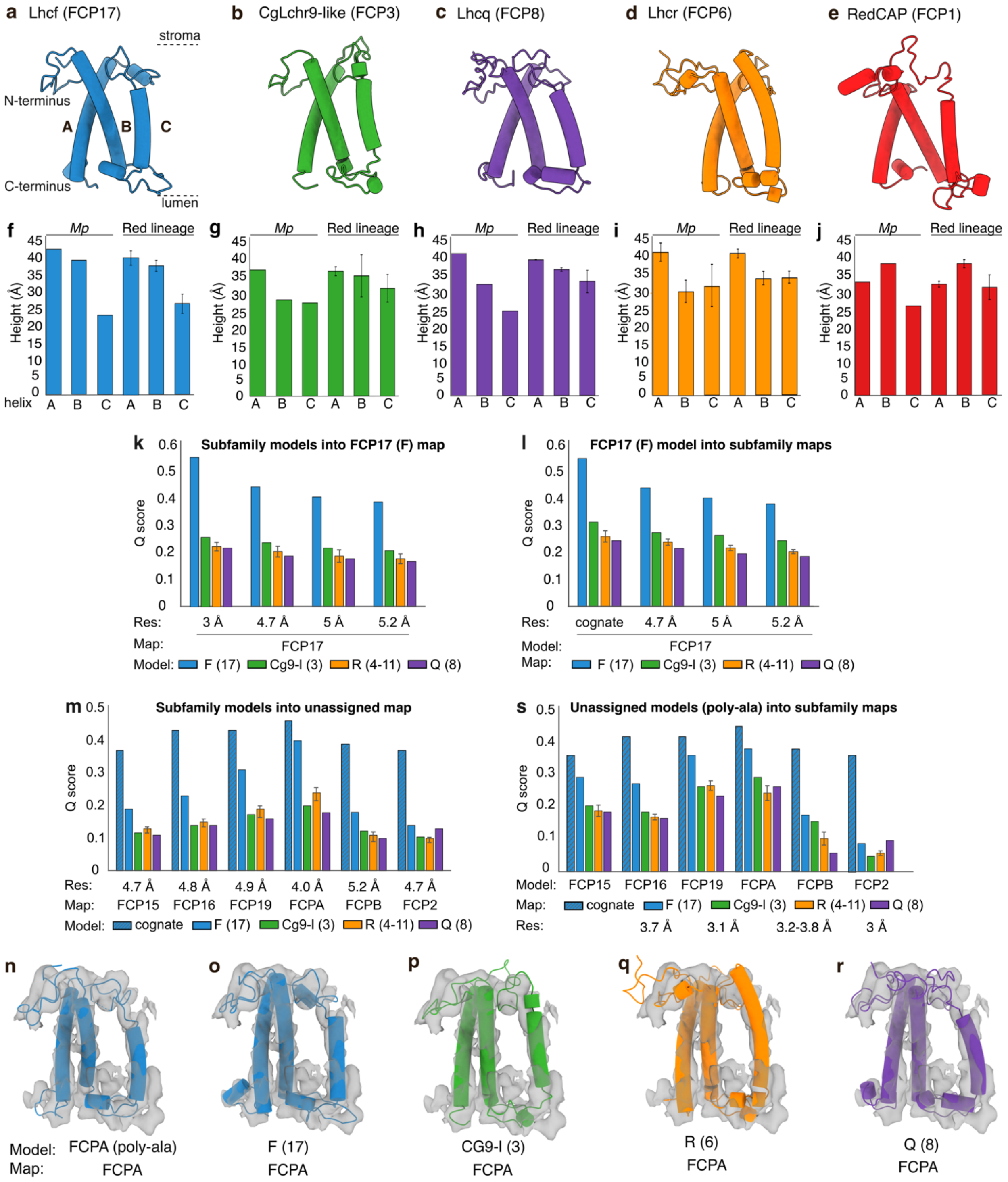

a Lhcf (FCP17)
b CgLchr9-like (FCP3)
c Lhcq (FCP8)
d Lhcr (FCP6)
e RedCAP (FCP1)
stroma
N-terminus
A
B
C
C-terminus
lumen
f
g
h
i
j
Mp
Red lineage
Height (Å)
helix
k Subfamily models into FCP17 (F) map
l FCP17 (F) model into subfamily maps
Q score
Res:
3 Å
4.7 Å
5 Å
5.2 Å
Map:
FCP17
Model: F (17) Cg9-l (3) R (4-11) Q (8)
cognate
Model:
FCP17
Map: F (17) Cg9-l (3) R (4-11) Q (8)
m Subfamily models into unassigned map
s Unassigned models (poly-ala) into subfamily maps
Res: 4.7 Å 4.8 Å 4.9 Å 4.0 Å 5.2 Å 4.7 Å
Map: FCP15 FCP16 FCP19 FCPA FCPB FCP2
Model: cognate F (17) Cg9-l (3) R (4-11) Q (8)
Model: FCP15 FCP16 FCP19 FCPA FCPB FCP2
Map: cognate F (17) Cg9-l (3) R (4-11) Q (8)
Res: 3.7 Å 3.1 Å 3.2-3.8 Å 3 Å
n
o
p
q
r
Model: FCPA (poly-ala)
Map: FCPA
F (17)
FCPA
CG9-l (3)
FCPA
R (6)
FCPA
Q (8)
FCPA

Supplementary Fig. 10. (previous page) **Structural characterization of *M. pyrifera*'s FCP subfamilies and subfamily assignment by map-model fit. a-e** Structures of each FCP subfamily shown in cartoon representation, coloured by subfamily. Helices A, B, C, N- and C-termini labelled in (a). RedCAP's helix F labelled in (c). Stroma and lumen marked with dashed lines. **f-j** Transmembrane helix heights for FCP helices A-C across subfamilies. Bar graphs show values for *M. pyrifera* ("*Mp*") and overall value across red lineage ("total", from *M. pyrifera*, *G. sulphuraria, C. placoidea, E.huxleyi* and *A. carterae*). For *M. pyrifera*, given that only Lhcr subfamily contains more than one representative, the average and standard deviation are only shown for Lhcr (n = 5-6). For the red lineage, values are averages of 2-22 individual helices across 2-4 available organisms as described in Methods. Error bars show standard deviation. Full dataset available in Supplementary Data 2. **k-s** Q-score validation on assigned FCPs (k, l) and test on unassigned FCPs (m-s). For clarity, representatives are labelled with the key letter for each family, followed by the FCP number, e.g., F (17) corresponds to subfamily Lhcf, using FCP17 as the representative. Subfamily colours used as in (a-j). Res, resolution. Given that only Lhcr subfamily contains more than one representative, the average and standard deviation are only shown for Lhcr (n = 6 for k; n = 5 for i and s; n = 7 for m). (k) Q-scores of subfamily models fit into FCP17 map at its cognate resolution (3 Å) and low-pass filtered to lower resolutions as indicated. (l) Q-scores of FCP17 model fit into subfamily representative maps at cognate (3.7, 3.1, 3.2-3.8, 3 Å for Lhcf, Cg9Lhcr-like, Lhcr, Lhcq respectively) and filtered to indicated lower resolutions. (m) Q-scores of subfamily representative models and cognate poly-alanine model (FCP15/16/19/A/B/2) fit into maps of FCP15/16/19/A/B/2. (n-r) Visual representation of model/map pairs in (m), illustrated with FCPA map. (s) Q-scores of poly-alanine models of FCP15/16/19/A/B/2 fit into maps of assigned subfamily representatives, as well as to their cognate maps (e.g., model for FCPA fit into FCPA map). Full dataset available in Supplementary Data 2.

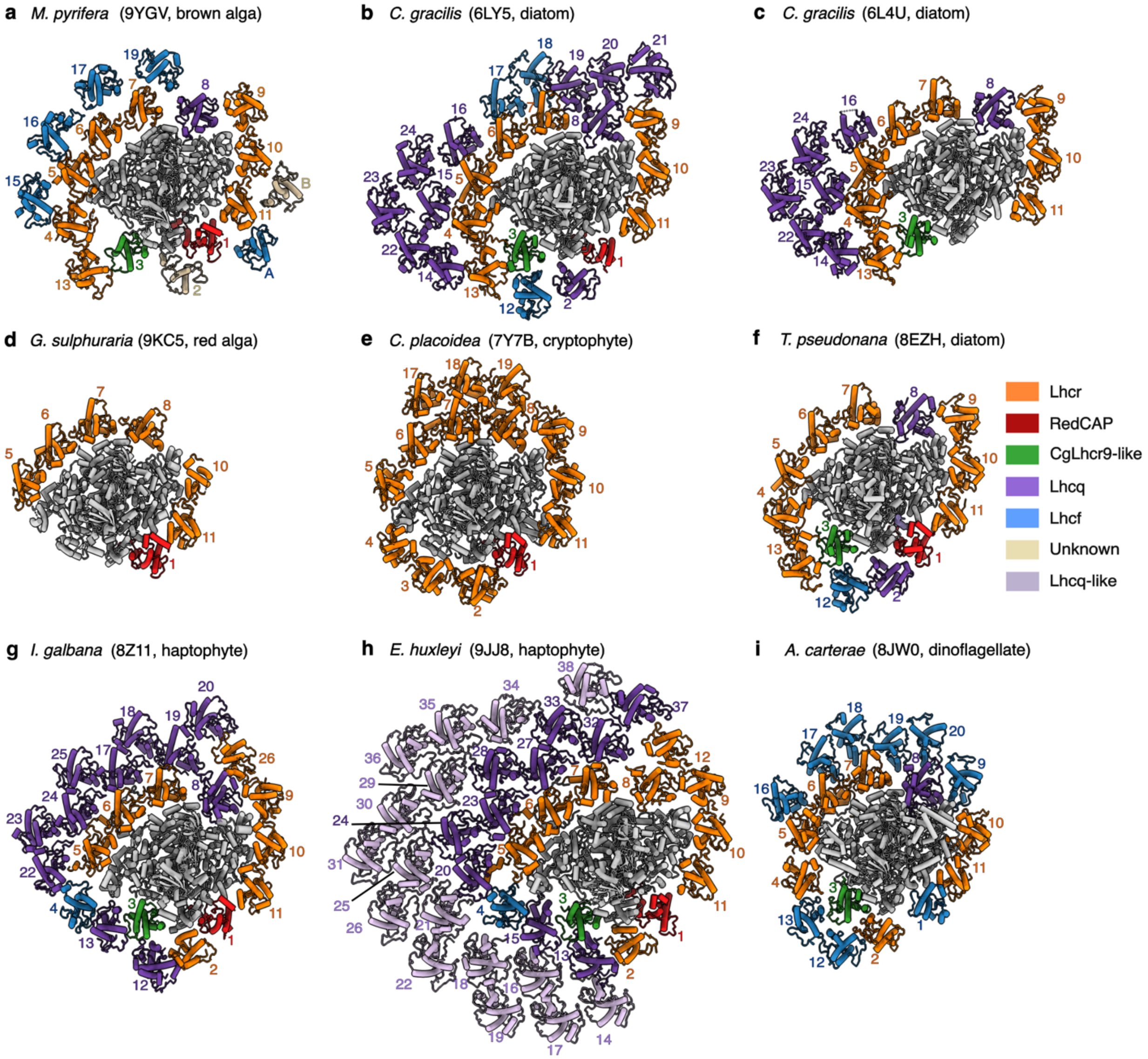

Supplementary Fig. 11. Comparison of FCP subfamilies in PSI antennae across red-lineage organisms. PSI-FCP supercomplexes from **a** brown alga *M. pyrifera* (PDB: 9YGV, this work), **b** diatom *C. gracilis* (PDB: 6LY5)[1], **c** diatom *C. gracilis* (PDB: 6L4U)[17], **d** red alga *G. sulphuraria* (PDB: 9KC5)[7], **e** cryptophyte *Croomonas placoidea* (PDB: 7Y7B)[11], **f** diatom *T. pseudonana* (PDB: 8ZEH)[18], **g** haptophyte *Isochrysis galbana* (PDB: 8Z11)[13], **h** haptophyte *Emiliania huxleyi* (PDB: 9JJ8)[14] and **i** dinoflagellate *Amphidinium carterae* (PDB: 8JW0)[15]. PDB codes shown next to species name. PSI shown in gray cartoon. FCP antenna shown in cartoon colored by subunit, according to FCP subfamily: Lhcr in orange, RedCAP in red, CgLhcr9-like in green, Lhcq in purple, Lhcf in blue, unknown in beige, Lhcq-like in lilac. FCP numbers used in this work shown, with FCP suffixes omitted for clarity.

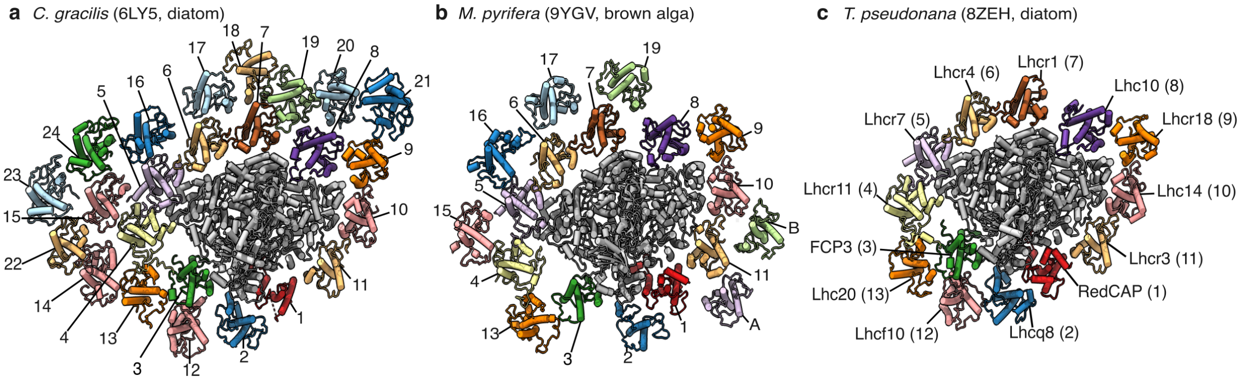

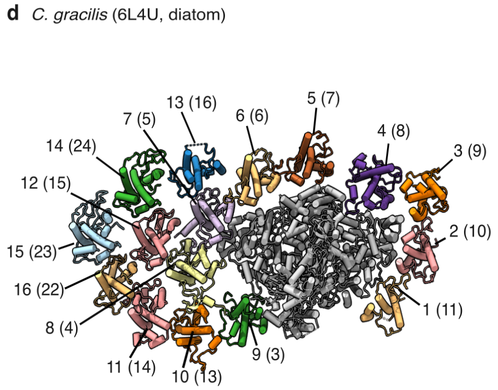

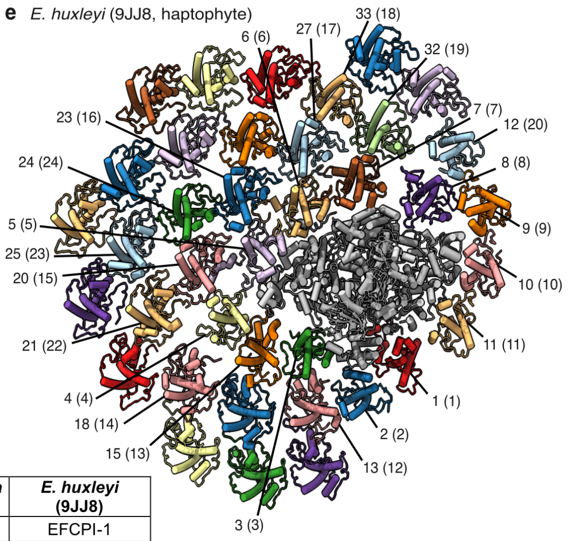

f

***M. pyrifera* (9YGV)**	***C. gracilis* (6LY5)**	***C. gracilis* (6L4U)**	***T. pseudonana* (8ZEH)**	***E. huxleyi* (9JJ8)**
FCP1	FCP1	-	RedCAP	EFCPI-1
FCP2	FCP2	-	Lhcq8	EFCPI-2
FCP3	FCP3	FCP9	FCP3	EFCPI-3
FCP4	FCP4	FCP8	Lhcr11	EFCPI-4
FCP5	FCP5	FCP7	Lhcr7	EFCPI-5
FCP6	FCP6	FCP6	Lhcr4	EFCPI-6
FCP7	FCP7	FCP5	Lhcr1	EFCPI-7
FCP8	FCP8	FCP4	Lhcr10	EFCPI-8
FCP9	FCP9	FCP3	Lhcr18	EFCPI-9
FCP10	FCP10	FCP2	Lhcr14	EFCPI-10
FCP11	FCP11	FCP1	Lhcr3	EFCPI-11
-	FCP12	-	Lhcf10	EFCPI-13
FCP13	FCP13	FCP10	Lhcr20	EFCPI-15
-	FCP14	FCP11	-	EFCPI-18
FCP15	FCP15	-	-	EFCPI-20
FCP16	FCP16	-	-	EFCPI-23
FCP17	FCP17	-	-	EFCPI-27
-	FCP18	-	-	EFCPI-33
FCP19	FCP19	-	-	EFCPI-32
-	FCP20	-	-	EFCPI-12
-	FCP21	-	-	-
-	FCP22	-	-	EFCPI-21
-	FCP23	-	-	EFCPI-25
-	FCP24	-	-	EFCPI-24
FCPA	-	-	-	-
FCPB	-	-	-	-

Supplementary Fig. 12. (previous page) **FCP antenna numbering and conversion. a-e** PSI-FCP supercomplexes from (a) *Chaetoceros gracilis* (PDB: 6LY5)[1], (b) *Macrocystis pyrifera* (PDB:9YGV), this work, (c) *Thalassiosira pseudonana* (PDB: 8ZEH)[18], (d) *C. gracilis* (PDB: 6L4U)[17], (e) *Emiliania huxleyi* (PDB: 9JJ8)[14]. PDB codes shown next to species name. PSI shown in grey cartoon. FCP antenna shown in cartoon coloured by subunit. Labels show nomenclature used in respective papers, with the number used in this work in parentheses. FCP suffixes omitted for clarity. **f** FCP nomenclature conversion from (a-e) in tabular form.

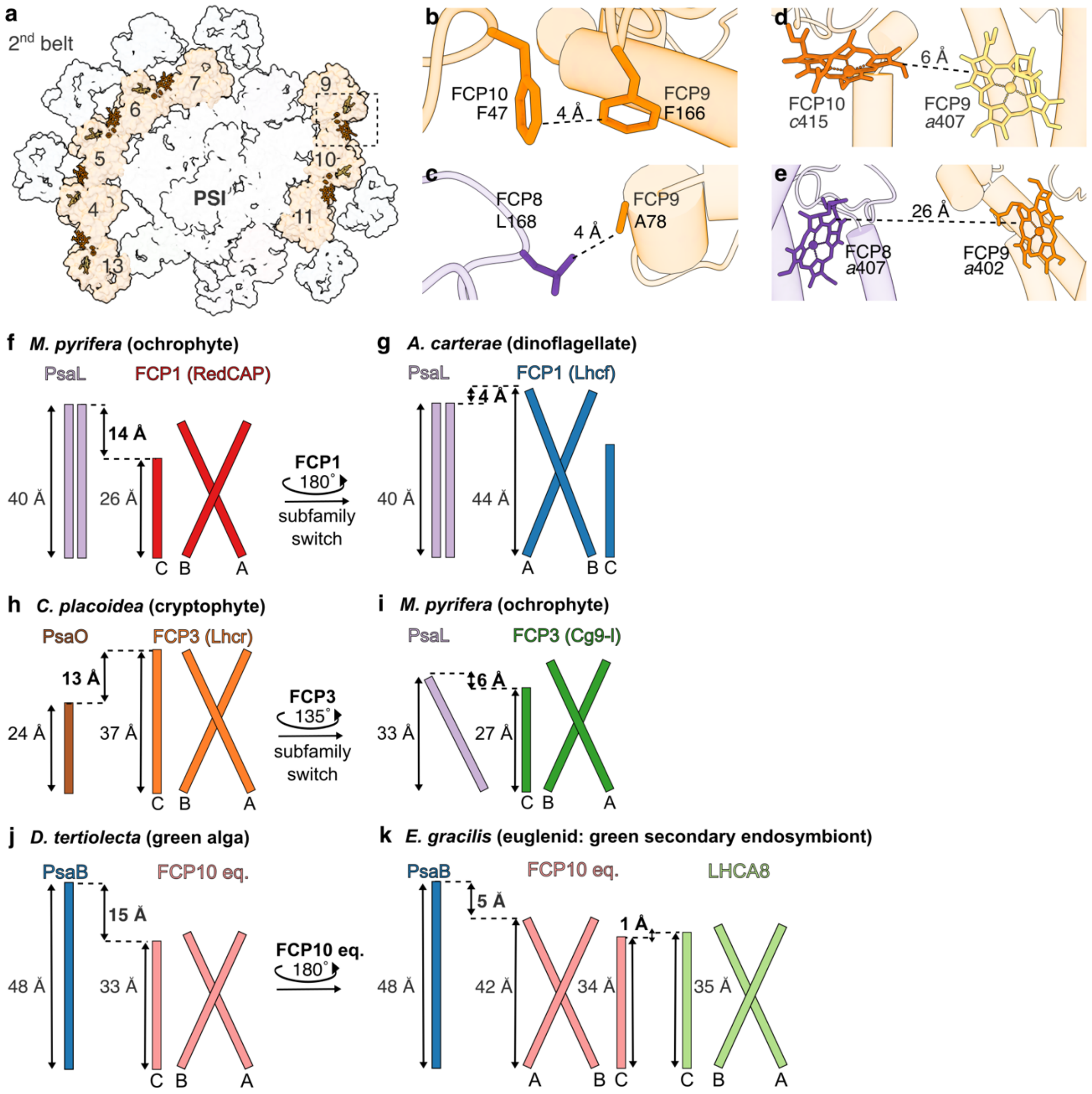

Supplementary Fig. 13. Characterization of *M. pyrifera* FCP interactions. a Stromal view of *M. pyrifera* PSI-FCP highlighting Lhcr features discussed in text. Lhcr subunits shown in orange surface and labelled with FCP number; core and second belt in grey surface. Porphyrin ring of key chlorophyll molecules and benzene ring of key phenylalanine residues shown in atom representation. Lhcr-specific features shown in dark orange; Lhcr chlorophyll found in other families shown in yellowish-orange. Dashed box indicates area detailed in (b-c). **b-e** Details of hydrophobic interactions (b-c) and chlorophyll-mediated energy transfer (d-e) between an Lhcr pair (b, d) or an Lhcr-non-Lhcr pair (c, e). Edge-to-edge distances between interaction partners indicated. **f-k** Schematic representation of FCP rotations and associated hydrophobic membrane thickness changes in red and green endosymbionts. FCP subunits coloured per subfamily in red lineage examples (RedCAP in red, Lhcf in blue, Lhcr in orange, CgLhcr9-like in green). Lack of neighbours to the right of FCP indicates absence of outer belt in that position in current structures. Helix thickness measurements in Å, see details in Supplementary Data 2. Thickness difference between neighbours in bold. (f, g) FCP1 rotation and subfamily switch between ochrophyte *M. pyrifera* (PDB: 9YGV, this work) and dinoflagellate *A. carterae* (PDB: 8JW0)[15]. (h, i) FCP3 rotation and subfamily switch between cryptophyte *C. placoidea* (PDB: 7Y7B)[11] and ochrophyte *M. pyrifera* (PDB: 9YGV, this work). (j, k) Rotation for FCP10-equivalent position in green lineage between green alga *Dunaliella tertiolecta* (PDB: 9MH1)[34] and green secondary endosymbiont *E. gracilis* (PDB: 9VJS)[35,36].

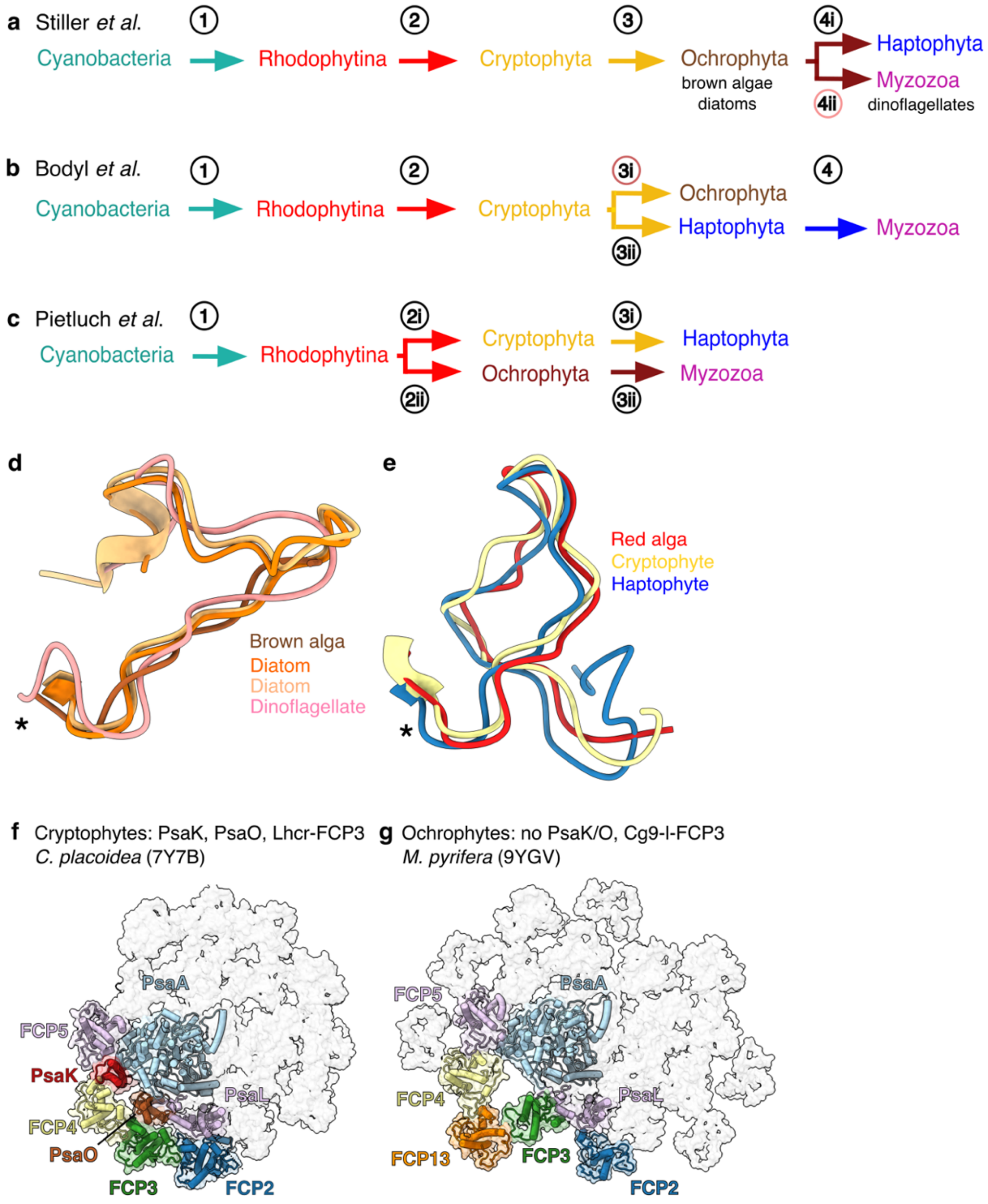

Supplementary Fig. 14. Structural support for red-lineage models of serial endosymbiosis. a-c Models of endosymbioses in the red lineage consistent with molecular timescale analysis[25,26] proposed by Stiller *et al*.[27] (a) Bodyl *et al*.[28] (b) and Pietluch *et al*.[26](c). Arrows and numbers represent serial and parallel endosymbioses. **d, e** Superposition of FCP9 N-terminal region of red algae (*P. purpureum*, PDB: 7Y5E)[8], cryptophytes (*C. placoidea*, PDB: 7Y7B)[11] and haptophytes (*I. galbana*, PDB: 8Z11)[13] or brown algae (*M. pyrifera*, PDB: 9YGV, this work), diatoms (*C. gracilis*, PDB: 6L4U)[17] and *T. pseudonana* (PDB: 8ZEH[18], as well as dinoflagellates (*A. carterae*, PDB: 8JW0)[15]. Note that FCP structures in (d, e) fully align in the region marked by an asterisk. **f, g** Overview of differences in the PsaK/L/O, FCP2/3/4 region between cryptophyte *C. placoidea* (f) and ochrophyte *M. pyrifera* (g), viewed from the stroma. Relevant subunits shown in coloured cartoon over semi-transparent surface of the whole supercomplex. PDB codes and references as in (d, e).

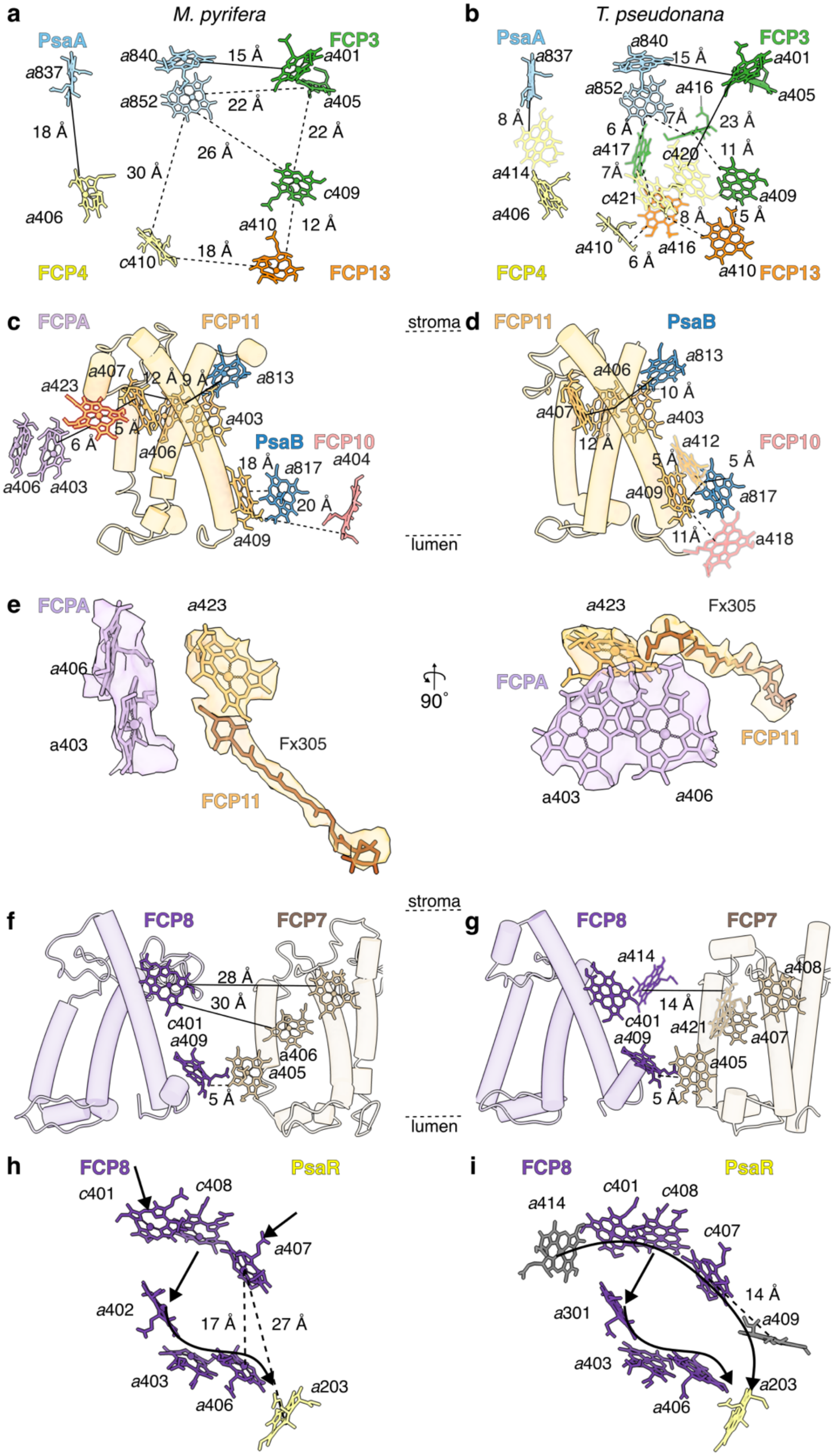

a
M. pyrifera
PsaA
FCP3
a840
15 Å
a401
a837
a852
22 Å
a405
18 Å
22 Å
26 Å
30 Å
c409
a406
a410
12 Å
c410
18 Å
FCP4
FCP13
b
T. pseudonana
PsaA
FCP3
a840
15 Å
a401
a837
a416
a405
a852
8 Å
7Å
23 Å
6 Å
a417
c420
11 Å
a414
7Å
a409
c421
8 Å
5 Å
a406
a410
a416
6 Å
a410
FCP4
FCP13
c
FCPA
FCP11
stroma
a407
a813
a423
12 Å
9 Å
a403
5 Å
6 Å
a406
PsaB
FCP10
18 Å
a817
a404
a406
a403
20 Å
a409
lumen
d
FCP11
PsaB
a813
a406
10 Å
a407
a403
12 Å
a412
FCP10
5 Å
5 Å
a409
a817
11Å
a418
e
FCPA
a423
a406
a403
Fx305
FCP11
90˚
a423
Fx305
FCPA
FCP11
a403
a406
f
stroma
FCP8
FCP7
28 Å
30 Å
c401
a409
a406
a405
5 Å
lumen
g
FCP8
FCP7
a414
a408
14 Å
c401
a421
a407
a409
a405
5 Å
h
FCP8
PsaR
c401
c408
a407
a402
17 Å
27 Å
a403
a203
a406
i
FCP8
PsaR
c401
c408
a414
c407
14 Å
a301
a409
a403
a203
a406

Supplementary Fig. 15. (previous page) **Full details for key differences in PSI-FCP chromophore arrangement between *M. pyrifera* and diatoms. a-d, f-g** Key chlorophyll differences between *M. pyrifera* (a, c, f) (PDB: 9YGV, this work) and *T. pseudonana* (b, d, g) (PDB: 8ZEH)[18] shown for various regions. Chromophores gained in *M. pyrifera* marked with thick brown border in *M. pyrifera* panels. Chromophores lost in *M. pyrifera* marked with grey border in *T. pseudonana* panels. Edge-to-edge distances between porphyrin rings indicated with solid lines for stromal connections and with dashed lines for lumenal connections. All chlorophylls labelled per position. (a, b) Differences in FCP3/4/13-PsaA. (c, d) Differences in FCP11/A-PsaB. **e** Map-model fit of chlorophyll *a* molecules modelled in *M. pyrifera* FCPA and its presumed transfer partner in FCP11. Fucoxanthin (Fx) molecule discussed in text also shown. Models coloured by subunit, map shown in semi-transparent surface coloured by subunit. Note that FCPA is absent in the available diatom structures. (f, g) Differences in FCP7/8. (h, i) Chlorophyll migration from FCP8 (*T. pseudonana* chl *a*405) to PsaR (*M. pyrifera* chl *a*205. **h-i** Stromal connections in the FCP8/9-PsaR pathways of *M. pyrifera* (h) and diatom *T. pseudonana* (i), viewed from the stroma. Chlorophyll molecules coloured by subunit. Diatom FCP8 chlorophyll molecules missing in *M. pyrifera* coloured in grey. Arrows denote predicted EET transfers.

				*Mp*His90
Rhodophyta		Bangiophyceae	*Galdieria yellowstonensis*	SAAYLISSVACLLIGSYCVYESNLLTP----LT-VSTINPLYVLGSLLVPYSWGLHVAG
			Galdieria sulphuraria	SAAYIISSVVCLLVGSYCVYESNLLSP----LT-VTTINPLYVLGSLLVPYSWGLHVAG
			Galdieria partita	SAAYVISSVICLLVGSYCVYESNLLSP----LT-VTSINPLFVLGSLLVPYSWGLHVAA
			Purpureofilum apyrenoidigerum	GSVFLMLSVIALFLGGYCVYQQNLNHL----LT-AASISPVYVVGSLLLPISWGCHVAA
			Porphyridium purpureum	SALYVLISIACFAIGVTSVAKSNLITP----LT-PESINPQYVVGSLLLPISWGAHTAA
		Rhodellophyceae	*Rhodella violacea*	GALFLALSVVSLGAGGYCCAESNIFNA----LT-PESVNPLFVAGSLLVPISWGCHVAA
		Stylonematophyceae	*Rhodosorus marinus*	GAFFLMMSVLCLFMGGYSVAESNLFDP----LT-AGTVKPLYVLGSLLLPISWGCHVAA
Crytophyta		Cryptophyceae	*Chroomonas placoidea*	SGNYIVASVVAAVVGAACTGQCIP-------LT--VSPNPVFILGSFLLPYSWALHVAA
			Hemiselmis virescens	SEQFLLASVLAVLIGAFSLSQAVP-------LK--PDPNPLFIAGSFMLPYSWALHVAA
			Hemiselmis andersenii	SEQFLLASVLAALIGAFSISQAVP-------LR--PDPNPLFIAGSFMLPYSWALHVGA
Haptophyta		Pavlovophyceae	*Pavlova gyrans*	SNVYAIASLGSLGVGLGCTAQANIFNV----LN-AENANPVIMAGSALTLYSFFLHIAC
		Prymnesiophyceae	*Diacronema lutheri*	SSVYAFTSLIALFVGVLCTAQSNILNV----LT-AESINPVLVLGSTLTLYSFFLHIAA
			Isochrysis galbana	SSIYGVTSAIAFSVGAYCIAQSNIINI----LS-GSTVNGFYVAGALLVPYSWGLHVAA
			Prymnesium parvum	SKFYGVTSGLALGIGLYCIAQSNLLNI----LS-GSSVNGFYVFGSLLVPYSWGLHVAS
			Phaeocystis globosa	SKFYGVTSGLALGIGLYCIAQSNLLNI----LS-GSSVNGFYVFGSLLVPYSWGLHVAS
Dinoflagellata		Dinophyceae	*Amphidinium carterae*	SNELAFLTMICLAIGAWGEAQFLWFDPQFAAIDRGEYFNPIYIIAASLLPVSFWAHIAC
			Polarella glacialis	SNELMYLSMLFFGIGLYGNLQFLYFDPQFPRVDSGLNFNVSYIVESLFLPMSFFFHIAC
			Effrenium voratum	SNELAFLSMLFFGVGLYGNLQFNYYDPQWAKVDAGGYFNVSYIVESLLLPISFFMHIAC
			Symbiodinium sp. CCMP2592	SNELAFLSMLFFGVGLYGNLQFNFYDPQWAKVDAGGFFNVSYIVESFLLPISFFMHIAC
Ochrophyta	Chrysista	Raphidophyceae	*Chattonella subsalsa*	SGILGPLSLVMFAAGGYCVHESNIFNI----LN-AETVKPLYVLGSLLTPISWGTHVAC
			Heterosigma akashiwo	SGILGPVSLAMLAAGTYCVHESNILNV----LN-AETVYPQYVLGSLLCPISWGTHVAA
		Pinguiophyceae	*Phaeomonas parva*	SGLLGPVSIISLLAGTYSVHESNILHT----LN-ADTVYPVFVIGSLLVPISWGLHVAA
		Phaeophyceae	***Macrocystis pyrifera***	SVGLGLVSLVAFAAGTYCIHTTNILHT----VS-PDSIQPIYVAGALLVPISWGLHVAS
			Ectocarpus siliculosus	SAGLGVVSLAAFAAGTYCIHETNILHT----VS-PDNIQPLYVAGALLVPISWGLHVAS
			Nemacystus decipiens	SAGLGIVSLAAFAAGTYCIHETNILHT----VS-PDTIQPLYVAGALLVPISWGLHVAS
			Cladosiphon okamuranus	SAGLGVISLVAFAAGTYCIHETNILHT----VS-PDNIQPLYVAGATLVPISWGLHVAS
			Undaria pinnatifida	SVGLGLVSLVAFAAGTYCIHTTNILHT----VS-PDSIQPLYVAGALLVPISWGLHVAS
			Saccharina japonica	SVGLGVVSLVAFAAGTYCIHTTNILHT----VS-PDSIQPIYVAGALLVPISWGLHVAS
			Saccharina latissima	SVGLGVVSLVAFAAGTYCIHTTNILHT----VS-PDSIQPIYVAGALLVPISWGLHVAS
		Xanthophyceae	*Vaucheria litorea*	SPALGVVSMIAFGIGTYCVHESNILHT----MT-VDTIYPKYIVGSLLTPISWGLHVAS
			Tribonema minus	SPALGVISLICLGVGTYSVHESNIFHT----LT-ADTINPGYIIGSLLTPVAWGTHVAS
		Chrysophyceae	*Ochromonadales sp. CCMP1393*	SGVFGPLSLVAFMVGTYCCHESNILNQ----IT-VDSINPLYVGGSALVPISWGMHVAA
			Ochromonadaceae sp. CCMP2298	SSIFGPLSLVALAVGTYCCHESNILNQ----IT-VTTIYPGYVAGSLLVPISWGMHVAA
	Diatomista	Pelagophyceae	*Pelagophyceae sp. CCMP2097*	SGLLAPLSVVSLAVGCYCVQESQLAAP----LS-AVTVNPLFIAGSLGVPISWGLHVAA
			Pelagomonadales sp. RCC1024	SALLGPVSGIFLMVGLYCVSASQLGAP----LT-AETLNPFYIAGSLCVPVSWGLHVAA
		Dictyophyceae	*Rhizochromulina marina*	SGLLGLTSLAFLAVGSYCAVQNNLFSL--------PDVNPLFVLGANLLPISWGLHVAS
			Florenciella parvula	SGLLGVVSGLFLGIGLYSAQANDLSSL--------PVVHWEYVVGANLLPISWGLHVAS
		Bolidophyceae	*Triparma laevis*	SGLLGLLSALSLGLCVYSCAQSGVLQQEPGAMSSGSWVKWYYVLGGFGGPMAWGMHVAS
			Triparma strigata	SGLLGLLSAASLGICVYSCAQSGVLQQEPGAMANGSWVKWYYVLGGFGGPLAWGMHVAS
		Diatomeae	*Thalassiosira oceanica*	SGTLAILSMMCLAACAYSCVQSGILLRVPGAVESGEWVKPEYILGGMAGPFAWGFHVAS
			Chaetoceros neogracilis	SGLLGLISIACAAVCVYSIAQSGVLQQQPGAYENGSWVKWYYVLGSFGGPLAWGTHVAS
			Cylindrotheca closterium	SGMLGIISLVLLGITISSCASSGALMQEPGAMANGSWVKPEYVLGSFMGPLAWGTHVAA
			Nitzschia inconspicua	SGMLGALSIMFLTICVYSCGQSFVLQQEPGALASGSWVKWYYVLGGFGGPLAWGTHVAA

Supplementary Fig. 16. PsaR alignment across ochrophyta. PsaR sequences from various ochrophytes with available genomes retrieved from NCBI GenBank, PhycoCosm and EukProt v3 databases, arranged by clade. Homologous position for His90 (coordinates PsaR-chl *a*205 in *M. pyrifera*) marked with red border. Chrysista clade marked in light brown box.

**Data collection and image processing**	
Microscope	Titan Krios
Voltage (kV)	300
Camera	Falcon 4i
Data collection software	EPU
Electron exposure ($e^-/Å^2$)	50
Exposure time (s)	4.95
Frame number	40
Defocus range (µm)	-1 to -2
Pixel size (Å)	1.217
Number of micrographs	15690
EMPIAR accession code	12998
Number of particles for final reconstruction	133,000
Symmetry imposed	C1
Map Resolution range (Å)	2.47-5.21
FSC threshold	0.143
**Model refinement statistics**	
Cross-correlation	
Mask	0.83
Volume	0.80
Model composition	
Non-hydrogen atoms	52,773
Protein residues	5,060
Ligands	318
Ramachandran	
Favored (%)	94.81
Allowed (%)	5.12
Outlier (%)	0.07
Rotamer outliers (%)	1.24
Clash score	6.08
RMSD	
Bond length (Å)	0.007
Bon angle (°)	1.557
B factors ($Å^2$, min/max/mean)	
Protein	3.41 / 281.00 / 72.63
Ligands	6.93 / 186.90 / 70.17
MolProbity score	1.76
PDB Accession code	9YGV

Supplementary Table 1. CryoEM data collection and atomic model statistics.

Map	EMDB code	Particles	Res. (Å)	Map entry title
Composite	76146	n.a.	2.47-5.21	Composite map of MpPSI-FCP supercomplex
Focused refined maps				
PSI	72805	133,000	2.47	Focused refinement of MpPSI-FCP around PSI
FCP1	72806	133,000	2.95	Focused refinement of MpPSI-FCP around FCP1
FCP2	72807	22,780	4.71	Focused refinement of MpPSI-FCP around FCP2
FCP3	72808	133,000	3.1	Focused refinement of MpPSI-FCP around FCP3
FCP4	72809	104,446	3.7	Focused refinement of MpPSI-FCP around FCP4
FCP5	72810	121,591	3.17	Focused refinement of MpPSI-FCP around FCP5
FCP6	72811	133,000	3.18	Focused refinement of MpPSI-FCP around FCP6
FCP7	72812	133,000	3.11	Focused refinement of MpPSI-FCP around FCP7
FCP8	72813	133,000	2.97	Focused refinement of MpPSI-FCP around FCP8
FCP9	72814	93,065	3.78	Focused refinement of MpPSI-FCP around FCP9
FCP10	72816	133,000	3.14	Focused refinement of MpPSI-FCP around FCP10
FCP11	72817	119,214	3.02	Focused refinement of MpPSI-FCP around FCP11
FCP13	72818	104,924	3.82	Focused refinement of MpPSI-FCP around FCP13
FCP15	72819	74,423	4.71	Focused refinement of MpPSI-FCP around FCP15
FCP16	72820	94,294	4.81	Focused refinement of MpPSI-FCP around FCP16
FCP17	72821	92,002	3.66	Focused refinement of MpPSI-FCP around FCP17
FCP19	72822	28,903	4.92	Focused refinement of MpPSI-FCP around FCP19
FCPA	75351	66,259	3.97	Focused refinement of MpPSI-FCP around FCPA
FCPB	72824	45,411	5.21	Focused refinement of MpPSI-FCP around FCPB

Supplementary Table 2. Summary of deposited maps. Details of composite and focused refined maps deposited in EMDB database. FCP, fucoxanthin-chlorophyll-*a*/*c*-protein; Mp, *Macrocystis pyrifera*; n.a., not applicable.

Name	Gene ID	Gene database	Loca-tion	Chain ID	Total res.	Modelled res.	Assignment confidence
**PsaA**	A0A8F0JYL2	UniprotKB	PSI	A	749	8-749	High
**PsaB**	A0A8F0F8K8	UniProtKB	PSI	B	734	4-733	High
**PsaC**	A0A8F0F8D0	UniProtKB	PSI	C	81	2-81	High
**PsaD**	A0A8F0F8I9	UniProtKB	PSI	D	132	7-131	High
**PsaE**	A0A8F0F8D1	UniProtKB	PSI	E	61	2-61	High
**PsaF**	A0A8F0JY91	UniProtKB	PSI	F	203	43-203	High
**PsaI**	A0A8F0F8C2	UniProtKB	PSI	I	36	2-35	High
**PsaJ**	A0A8F0F8B4	UniProtKB	PSI	J	42	1-41	High
**PsaL**	A0A8F0F8N6	UniProtKB	PSI	L	145	2-143	High
**PsaM**	A0A8F0JYJ8	UniProtKB	PSI	M	30	1-30	High
**PsaR**	CAM9152197.1	GenBank	PSI	R	132	48-132	High
**FCP1**	9470359	PhycoCosm	1st	1	231	54-231	High
**FCP2**	unassigned	n.a.	1st	2	n.a.	n.a.	n.a.
**FCP3**	9638407	PhycoCosm	1st	3	191	27-181	High
**FCP4**	DN157149	PhycoCosm	1st	4	237	40-235	Intermediate
**FCP5**	9353087	PhycoCosm	1st	5	222	44-218	High
**FCP6**	9608268	PhycoCosm	1st	6	212	39-211	High
**FCP7**	DN156408	PhycoCosm	1st	7	169	1-169	High
**FCP8**	9480344	PhycoCosm	1st	8	215	40-214	High
**FCP9**	9541138	PhycoCosm	1st	9	216	62-208	Intermediate
**FCP10**	9608268	PhycoCosm	1st	10	212	40-211	High
**FCP11**	9689568	PhycoCosm	1st	11	213	45-211	High
**FCP13**	9541138	PhycoCosm	2nd	13	216	66-170, 176-214	Intermediate
**FCP15**	unassigned	n.a.	2nd	15	n.a.	n.a.	n.a. (Lhcf)
**FCP16**	unassigned	n.a.	2nd	16	n.a.	n.a.	n.a.(Lhcf)
**FCP17**	1228943	PhycoCosm	2nd	17	218	46-149, 164-205	Intermediate
**FCP19**	unassigned	n.a.	2nd	19	n.a.	n.a.	n.a. (Lhcf)
**FCPA**	unassigned	n.a.	2nd	a	n.a.	n.a.	n.a. (Lhcf)
**FCPB**	unassigned	n.a.	2nd	b	n.a.	n.a.	n.a.

Supplementary Table 3. Model statistics by subunit. *M. pyrifera* PSI-FCP supercomplex model details by subunit. For unassigned subunits, parentheses indicate best-matching FCP subfamily. PQN, phylloquinone; SF4, [4Fe-4S] cluster; CLA, chlorophyll *a*; KC1, CLA, chlorophyll *c*; A86, fucoxanthin; XAT, violaxanthin; BCR, beta-carotene; DGD, digalactosyl-diacyl-glycerol; LHG, di-palmitoyl-phosphatydil-glycerole; LMG, distearoyl-monogalactosyl-diglyceride; n.a., not available; 1st, first belt; 2nd, second belt; res., residues.

Subunit name	PQN	SF4	CLA	KC1	A86	XAT	BCR	DGD	LHG	LMG
**PSI**										
PsaA	1	1	44	-	1	-	5	-	5	-
PsaB	1	-	40	-	1	-	6	-	2	-
PsaC	-	2	-	-	-	-	-	-	-	-
PsaD	-	-	-	-	-	-	-	-	-	-
PsaE	-	-	-	-	-	-	-	-	-	-
PsaF	-	-	4	-	-	-	2	-	-	-
PsaI	-	-	1	-	-	-	2	-	1	-
PsaJ	-	-	1	-	-	1	1	-	-	1
PsaL	-	-	3	-	-	-	2	-	-	-
PsaM	-	-	-	-	-	-	1	-	-	-
PsaR	-	-	2	-	1	-	1	1	-	-
Total PSI	2	3	95	0	3	1	20	1	8	1
**FCP**										
FCP1	-	-	7	1	2	1	1	-	1	-
FCP2	-	-	-	-	-	-	-	-	-	-
FCP3	-	-	6	3	3	1	-	-	-	-
FCP4	-	-	7	4	2	2	-	1	-	-
FCP5	-	-	8	1	1	3	-	1	-	-
FCP6	-	-	8	4	1	4	-	-	1	-
FCP7	-	-	9	2	-	5	-	-	1	-
FCP8	-	-	7	2	1	2	1	-	-	-
FCP9	-	-	7	2	1	1	-	-	1	-
FCP10	-	-	9	3	2	3	-	-	2	-
FCP11	-	-	9	1	1	1	-	-	1	-
FCP13	-	-	6	1	-	2	-	-	-	-
FCP15	-	-	1	-	-	-	-	-	-	-
FCP16	-	-	5	-	-	-	-	-	-	-
FCP17	-	-	7	2	1	1	1	-	-	-
FCP19	-	-	3	-	-	-	-	-	-	-
FCPA	-	-	5	1	-	-	-	-	-	-
FCPB	-	-	-	-	-	-	-	-	-	-
**Total FCP**	0	0	104	27	15	26	3	2	7	0
**Total PSI-FCP**	2	3	199	27	18	27	23	3	15	1

Supplementary Table 3 (continued) **Model statistics by subunit.** *M. pyrifera* PSI-FCP supercomplex model details by subunit. PQN, phylloquinone; SF4, [4Fe-4S] cluster; CLA, chlorophyll *a*; KC1, CLA, chlorophyll *c*; A86, fucoxanthin; XAT, violaxanthin; BCR, beta-carotene; DGD, digalactosyl-diacyl-glycerol; LHG, di-palmitoyl-phosphatydil-glycerole; LMG, distearoyl-monogalactosyl-diglyceride; n.a., not available.

Site	FCP1	FCP2	FCP3	FCP4	FCP5	FCP6	FCP7	FCP8	FCP9	FCP10
301	-	-	Fx313	-	-	Vx321	Vx322	-	-	Fx326
302	Fx301	-	Fx314	Vx340	Vx321	Vx325	Vx323	Vx319	Fx315	Vx321
303	Fx318	-	Vx317	Vx343	Vx312	Vx324	Vx321	Vx318	Vx317	Vx323
304	-	-	-	-	-	-	-	-	-	-
305	Bcr317	-	Fx311	Fx335	Fx322	Fx323	Vx320	Fx311	-	Vx322
306	-	-	-	Fx338	-	Vx320	Vx324	-	-	Fx319
307	-	-	-	-	Vx320	-	-	Bcr315	-	-
308	Vx316	-	-	-	-	-	-	-	-	-
401	-	-	*a*307	*c*326	*c*316	*a*307	*a*308	*c*306	-	*a*309
402	*a*305	-	*a*301	*a*321	*a*302	*a*301	*a*301	*a*301	*a*301	*a*303
403	*a*312	-	*c*302	*a*322	*a*303	*a*302	*a*302	*a*302	*a*302	*a*304
404	-	-	*a*303	*a*323	*a*304	*a*303	*a*303	*a*303	*a*303	*a*305
405	*a*313	-	*a*304	-	*a*306	*a*304	*a*318	-	*c*304	*c*306
406	*a*308	-	*a*305	*a*324	*a*305	*c*305	*a*306	*a*304	*a*305	*a*307
407	*a*309	-	*a*306	*a*325	*a*324	*a*306	*a*307	*a*305	*a*306	*a*308
408	*c*310	-	*c*308	*c*327	*a*318	*c*308	*c*309	*c*307	*c*308	*a*310
409	*a*300	-	*c*309	*a*328	-	*c*309	*a*310	*a*320	*a*309	*a*311
410	*a*315	-	-	*c*331	-	*a*311	-	-	*a*310	*a*325
412	-	-	-	-	-	*a*322	*a*319	-	-	*c*314
413	-	-	-	-	-	-	-	*a*316	-	-
414	-	-	-	*c*330	-	-	-	-	-	-
415	-	-	-	*a*341	*a*310	*c*310	*c*311	-	-	*c*312
416	-	-	-	-	-	-	-	-	-	-
417	-	-	-	-	-	-	-	-	-	-
419	-	-	-	-	-	-	-	-	-	-
420	-	-	-	-	-	-	-	-	-	-
421	-	-	-	-	-	-	-	-	-	-
422	-	-	-	-	-	-	-	-	-	-
423	-	-	-	-	-	-	-	-	-	-

Supplementary Table 4. Correspondence between chromophore PDB numbering and binding site numbering. Binding site numbering per [18]. *a*, chlorophyll *a*; *c*, chlorophyll *c*; Bcr, beta-carotene; Fx, fucoxanthin; Vx, violaxanthin.

Site	FCP11	FCP13	FCP17	FCP15	FCP16	FCP19	FCPA	FCPB
301	-	-	-	-	-	-	-	-
302	-	Vx317	Fx310	-	-	-	-	-
303	Vx316	Vx316	Vx313	-	-	-	-	-
304	-	-	-	-	-	-	-	-
305	Fx309	-	Bcr312	-	-	-	-	-
306	-	-	-	-	-	-	-	-
307	-	-	-	-	-	-	-	-
308	-	-	-	-	-	-	-	-
401	*a*305	-	*a*304	-	-	-	*a*299	-
402	*a*301	*a*301	*c*319	-	*a*297	*a*261	*a*300	-
403	*a*302	*a*302	*a*298	-	*a*298	*a*262	*a*301	-
404	*a*312	*a*303	*a*299	-	-	-	-	-
405	-	*c*304	*a*311	*a*272	-	-	*a*303	-
406	*a*303	*a*305	*a*302	-	-	-	*a*304	-
407	*a*304	*a*306	*a*303	-	*a*302	*a*267	-	-
408	*a*315	-	*c*305	-	*a*305	-	*c*306	-
409	*a*307	-	*a*306	-	*a*304	-	-	-
410	-	*a*310	-	-	-	-	-	-
412	-	-	-	-	-	-	-	-
413	-	-	-	-	-	-	-	-
414	-	-	-	-	-	-	-	-
415	*c*308	-	-	-	-	-	-	-
416	-	-	-	-	-	-	-	-
417	-	-	-	-	-	-	-	-
419	-	-	-	-	-	-	-	-
420	-	-	-	-	-	-	-	-
421	-	-	-	-	-	-	-	-
422	-	-	-	-	-	-	-	-
423	*a*311	-	-	-	-	-	-	-

Supplementary Table 4 (continued)**. Correspondence between chromophore PDB numbering and binding site numbering.** Binding site numbering per [9]. *a*, chlorophyll *a*; *c*, chlorophyll *c*; Bcr, beta-carotene; A86, fucoxanthin; Vx, violaxanthin.

	Red algae	Red algae	Crypto-phytes	Hapto-phytes	Diatom (ochro-phyte)	Diatom (ochro-phyte)	Brown algae (ochro-phyte)	Dino-flagellate (myzoza)	Supports Stiller (A), Bodyl (B) or Pietluch (C) [26–28]	Comments
**Representative species**	***G. sulphuraria***	***P. purpureum***	***C. placoidea***	***I. galbana***	***T. pseudonana***	***C. gracilis***	***M. pyrifera***	***A. carterae***		
**PDBs used**	9KC5	7Y5E	*7Y7B*	8Z11, 9JJ8	8XLS, 8ZEH	6LY5, 6L4U	9YGV	8JW0		
**Other PDBs**		5ZGB, 5ZGH, 6FOS	8WM6, 8WMJ, 8WMW		8ZET			8JZE, 8JZF		
**References**	[7]	[8–10]	[11,12]	[13,14]	[2,18]	[1,17]	This work	[15]		
**LHC families**	R, RedCAP	R, RedCAP	R, RedCAP, Z	R, Red, Z, X, Cg9, Q, F					A	[6,37]
**FCP1 family**	RedCAP	RedCAP	RedCAP	RedCAP	RedCAP	RedCAP	RedCAP	LhcF	A, B, C	Gene loss of RedCAP enables FCP1 switch
**RedCAP gene present**	Yes	Yes	Yes	Yes	Yes	Yes	Yes	No		
**FCP1 standard orientation**	Yes	Yes	Yes	Yes	Yes	Yes	Yes	No, 180°		
**FCP1 #EET to PSI**	1	1	1	1	1	1	1	4		
**PsaL encoding**	Plastid	Plastid	Plastid	Plastid	Plastid	Plastid	Plastid	Nuclear	A, B, C	
**PsaK present**	Yes	Yes	Yes	Yes	No	No	No	No	B, C	
**PsaK encoded**	Plastid	Plastid	Plastid	Nucleus	n. a.	n. a.	n. a.	n. a.		
**FCP4 family**	n.a. (R)	R	R	F	R	R	R	R	A, C	For B, dino would have to back-replace FCP4-Lhrf into Lhcr

**PsaO present**	Yes	Yes	Yes	No	No	No	No	No	A	PsaO needs to have been lost 1x in A, 2x in B, 2x in C
**PsaO encoded**	Nucleus	Nucleus	Nucleus	n. a.	n. a.	n. a.	n. a.	n. a.		Gene loss of PsaO enables FCP3 switch
**FCP3 family**	n. a.(R)	n. a.(R)	R	Cg9-l	Cg9-l	Cg9-l	Cg9-l	Cg9-l	A	Or B, if engulfed cryptophyte had already lost PsaO
**FCP3 standard orientation**	n. a.	n. a.	Yes	No, 135°	No, 135°	No, 135°	No, 135°	No, 135°		
**FCP3 main core partner**	n. a.	O	O	L	L	L	L	L		
**FCP2 family**	n. a. (R)	n. a. (R)	R	R	Q	Q	Q (t.b.c.)	R	B	
**Prominent FCP3/4/13 EET**	n. a.	No	No	No	Yes	Yes	No	No		Diatom-specific
**FCP 4-5 LhcR aromatic connection**	n. a.	n. a.	Yes	No	Yes	Yes	No	No		Discrepancy within ochrophyta
**FCP 8/R pathway chl 316**	n. a.	Yes	Yes	Yes	No, but new chl a **319**	No	Yes	Yes		Discrepancy within ochrophyta and diatoms
**FCP8/R pathway chl 312**	No	No	No	Yes	Yes	Yes	No, but new chl **205** (PsaR-His90)	No		Brown-algae-specific pathway
**FCP9 family**	n. a.	R	R	R	R	R	R	F	A, B, C	
**FCP9 chl 301 R-family SxS/A I/L P**	n. a.	Yes	Yes	Yes	No	Yes	Yes	No		Discrepancy within ochrophyta and diatoms
**FCP9 R-specific chl present**	n. a.	Yes	Yes	Yes	No	No	No	No (FCP9 is F)		
**FCP9 N-ter structure**	n. a.	Towards core	Towards core	Towards core	Towards periphery	Towards periphery	Towards periphery	Towards periphery	C	
**FCP13 family**	n. a.	R	R	Q	R	R	R	F	C	

**FCP11 chl 311 gain**	No	No	No	No	No	No	Yes	No		Kelp-specific, likely related to energy transfer from novel FCPA/B
**Inner belt family majority**	R	R	R	R	R	R	R	R		Strong conservation
**Outer belt family majority**	n. a.	n. a.	R	Q	R/F	Q	F (t.b.c.)	F	C	Strong diversification, even within diatoms

Supplementary Table 5. Summary of differences between red-lineage clades. Summary of clade differences in protein and chromophore features discussed in text. FCP subfamilies represented by the last letter, e.g., R, Lhcr. Cg9-l, CgLhcr9-like; n.a., not applicable; t.b.c., to be confirmed.

Supplementary Note 1:

M. pyrifera PSI-FCP composition and structure

Our model contains 3 [4Fe-4S] clusters, 2 phylloquinones, 199 chl *a*, 27 chl *c*, 23 β-carotenes, 26 violaxanthins, 18 fucoxanthins and 19 lipids (Supplementary Fig. 2, Supplementary tables S3-S4). We detected and quantified the pigments chlorophyll *a*, chlorophyll *c*, fucoxanthin, violaxanthin, zeaxanthin and β-carotene using high performance liquid chromatography (Supplementary Fig. 2d).

Inspection of the plastocyanin, cytochrome c_6 and ferredoxin binding regions of PSI revealed almost complete conservation (see below). We did not find evidence for stromal subunit PsaS/Psa29, which was recently reported in diatoms[1,2], in either the cryoEM density or the *M. pyrifera* genome[3]. This supports the notion that PsaS/Psa29 is a Diatomista innovation[2].

All FCP subunits showed the expected topology (transmembrane helices B, C, A) and Arg-Glu salt bridges between helices A and B seen in all photosynthetic organisms so far[4,5] (Supplementary Fig. 7a). Chromophore-binding motifs identified in diatoms for each FCP subfamily are also conserved in *M. pyrifera*[6] (Supplementary Fig. 7b-e). As seen in other red-lineage organisms, FCP interactions were mediated mostly on the stromal side, by FCPs' N-terminal loop and helix C regions (FCP:PSI, Supplementary Fig. 7f, g), or N-terminal loop and C-A loop (FCP:FCP, Supplementary Fig. 7h, i)[7–16,1,17,2,18].

Structural analysis of *M. pyrifera*'s PSI and predicted electron donors suggest a Type-III mechanism of electron transfer

PSI receives electrons from soluble electron donors that bind on the lumenal surface. These electrons reduce the photo-oxidised special pair P700 at the reaction center and are then transferred through cofactors to ferredoxin, which binds on the PSI stromal domain. The electron donor interacts with the N-terminal α1 helix of PsaF *via* long-range interactions before docking at the PsaA/B interface (Supplementary Fig. 7j-l). Two families of electron donors have been described for PSI: plastocyanin (Pc), a copper-binding protein widely used in the green lineage, and cytochrome c_6 (cyt c_6), a *c*-type heme protein typically found in cyanobacteria and the red lineage[19]. Pc appears to have been lost in most red algae and the red lineage[19]. However, Pc has been seen to act as an alternative PSI donor under iron limitation in certain diatoms[20]. The *M. pyrifera* genome encodes both Pc and cyt c_6, raising questions about the preferred electron donor and transfer mechanism to PSI. To examine how Pc and cyt c_6 might interact with PSI, we identified their coding sequences in the *M. pyrifera* genome, generated structural-prediction models and docked the models onto *M. pyrifera*'s PSI.

As seen in sequence alignments and electrostatic-surface analysis, similar to plants, green algae and diatoms, the lumenal region of *M. pyrifera*'s PsaF possesses a positively charged insertion between α1 and α2 helices relative to cyanobacteria (Supplementary Fig. 7m, n). In *M. pyrifera*, both Pc (gene ID: DN119526 c0 g1 i1) and cyt c_6 (gene ID: DN39228 c0 g1 i3) display conserved acidic patches that complement this positively charged region (Supplementary Fig. 7o-q). Moreover, unbiased docking of predicted structural models of *M. pyrifera* Pc and cyt c_6 to PSI positioned their cofactors within ~15 Å (Pc) and ~17 Å (cyt c_6) of the reaction center (Supplementary Fig. 7r, s), with predicted binding energies of -5.7 kcal/mol (Pc) and -9.2

kcal/mol (cyt c_6). These docking results resemble the hydrophobic docking interface to PsaA/PsaB subunits, the distance to the P700 special pair, and the orientation of the negatively charged donor surfaces toward the α1 helix of PsaF, as described in high-resolution structures of PSI-donor complexes from cyanobacteria and plants, as well as in diatom *in silico* models[21,22]. The stronger interaction of *M. pyrifera*'s cyt c_6 relative to Pc is consistent with the cyt c_6 preference reported in diatoms, suggesting that brown algal PSI predominantly uses cyt c_6 while maintaining the capacity to interact with Pc[22]. Moreover, the electrostatic complementarity between the basic patch on PsaF and the acidic surfaces of Pc and cyt c_6 suggests that, like most eukaryotic phototrophs, *M. pyrifera* uses a long-range, electrostatics-based Type-III mechanism for electron transfer to PSI, rather than Type I or II collision-based mechanism described in cyanobacteria.

Stromal domain: implications for ferredoxin (Fd) binding

PSI transfers electrons to ferredoxin (Fd), which then acts as a central reducing agent in chloroplasts, including the reduction of $NADP^+$ to NADPH by FNR[23]. The PSI:Fd electron transfer is enabled by PsaC's 4Fe4S clusters. In previously studied organisms, the protein:protein interactions between PSI and Fd are mediated by electrostatic interactions with PsaA/C/D/E [24]. Upon examination of the *M. pyrifera* stromal domain, we determined that all reported residues mediating the PSI:Fd interaction are conserved, except for a substitution of Arg to Lys in *Mp*PsaE-K4 and the loss of Lys to Thr in *Mp*PsaD-T105, both of which are conserved in other brown algae[24] (Supplementary Fig. 7t).

The recent diatom PSI structures revealed the presence of an additional subunit (PsaS, a.k.a. Psa29) in the stromal domain[1,2]. Diatom PsaS interacts mainly with PsaC and PsaD, with some additional minor interactions with stroma-facing regions of PsaB/I/L. Given that PsaS does not contain redox cofactors and that its binding site is ~14 Å away from Fd at its closest, it is unlikely that PsaS has direct effects on Fd recruitment or electron transfer. Rather, PsaS may serve to stabilise PSI's stromal domain or to indirectly regulate the PSI:Fd interaction. In contrast to diatoms, we did not find evidence for a PsaS homologue in the *M. pyrifera* cryoEM density, even after specific masked classifications and focused refinements of this area using a diatom PsaS models[1,2]. We also failed to find homologues PsaS using diatom PsaS queries in homologue searches in *M. pyrifera*, *E. siliculosus* or Phaecyacea at large. This is in line with the finding[2] that PsaS is absent in Bolidophyceae, a sister group to diatoms. Further, it suggests that PsaS is a diatom innovation not present in the common ancestor of Ochrophyta. By extension, this implies that Chrysista and Diatomista clades use different mechanisms to regulate electron transfer from PSI to Fd. Evaluation of electron transfer in the presence and absence of PsaS is needed to test this hypothesis.

Supplementary Note 2:

Implications for the endosymbiotic origin(s) of the red lineage

The endosymbiotic relationships between the phyla of the red lineage remain unclear, with three models consistent with recent molecular-timescale-constrained phylogenetic analyses[25,26] (Supplementary Fig. 14a-c). A model from Stiller *et al.* posits serial endosymbioses of red algae to cryptophytes to ochrophytes to the ancestor of haptophytes and myzozoa (dinoflagellates)[27] (Supplementary Fig. 14a). In contrast, Bodyl *et al.* propose serial endosymbioses from red algae

to cryptophytes to the ancestor of ochrophytes and haptophytes, followed by an engulfment of a haptophyte to give rise to myzozoa[28] (Supplementary Fig. 14b). Additionally, Pietluch *et al.* propose that there were two separate secondary endosymbioses of red algae followed by separate tertiary endosymbioses from cryptophyte to haptophyte and from ochrophyte to myzozoa[26] (Supplementary Fig. 14c). Comparing the structures and composition of photosystems provides a complementary approach to evaluate evolutionary relationships[29]. Given the different roles of ochrophytes in the three models, examining brown algal and diatom structures is particularly helpful.

The Pietluch model is strongly supported by the FCP9/13 structures, which show two distinct clusters: cryptophytes/haptophytes and ochrophytes/dinoflagellates[26] (Supplementary Fig. 14c-e). These two Lhcr subunits lack the otherwise Lhcr-specific chl 415 in *M. pyrifera*. The main driver of the chlorophyll loss is the fact that their N-terminal loop adopts a conformation unlike that seen in the rest of the Lhcr subunits. In *M. pyrifera*'s FCP9/13, the N-ter loop travels away from the core rather than forming a core-facing loop with the chlorophyll-binding turn (Supplementary Fig. 14d). An analogous situation in seen in diatoms and dinoflagellates. In the latter, dinoflagellate FCP9/13 belong to the Lhcf subfamily, with a short 15-amino-acid helix C, but otherwise fully align with *M. pyrifera*'s FCP9/13 structure. In contrast, FCP9/13 contain the traditional Lhcr N-terminal motif and structure in red algae, cryptophytes and haptophytes (Supplementary Fig. 14e).

The pattern of inheritance and protein arrangement in the PsaK/O-FCP2/3 region provide additional cases to differentiate the models (Supplementary Fig. 14f, g). PsaO is encoded in the nucleus of red algae and of cryptophytes and is absent in the other red phyla[30,31,13,15,16,1,17,2]. The pattern suggests that red algal PsaO was transferred to the nucleus of the ancestral cryptophyte upon secondary endosymbiosis and was lost in subsequent endosymbioses due to failure to transfer to the nucleus from the engulfed nucleomorph. This implies PsaO's nuclear-transfer failure happened once for the Stiller model, twice for the Bodyl model and twice for the Pietluch model (Supplementary Fig. 14a-c). Moreover, the loss of PsaO from PSI is perfectly correlated with the subfamily switch and physical rotation of FCP3 (Fig. 2g-l, Supplementary Fig. 11). The facts that ochrophyte, haptophyte and dinoflagellate show the same new FCP3 subfamily (Cg9-l) and high similarity in FCP3 structure, orientation and PSI-binding, and that these clades are in stark contrast to red algae and cryptophytes, is most parsimoniously explained by the Stiller model[27] (Fig. 2g, h, Supplementary Fig. 11, 14a, f, g). The second case of subfamily and rotational switch, i.e., FCP1's RedCAP-to-Lhcf change, does not provide evidence to disambiguate the three models, as the modification occurred in dinoflagellates, i.e., upon the terminal endosymbiosis in all models (Supplementary Fig. 14a-c).

Lastly, PsaK is encoded in the plastid of red algae, cryptophytes and in the nucleus of haptophytes[30–32]. This is consistent with a nuclear transfer from the cryptophyte nucleomorph to the haptophyte nucleus in the Bodyl and Pietluch models (Supplementary Fig. 14b,3ii; 14c,3i), with a subsequent nuclear-transfer failure from haptophytes to dinoflagellates in the Bodyl model (Supplementary Fig. 14b,4). In the Stiller model, the PsaK pattern implies a nuclear-transfer failure from cryptophyte to ochrophyte (Supplementary Fig. 14a,3) and then re-gain from ochrophyte to haptophyte (Supplementary Fig. 14a,4i), or other non-parsimonious scenarios[27]. FCP sub-family changes in FCP2 in the vicinity of PsaK/O are also more consistent with the

Bodyl model: FCP2 is an Lhcr protein in cryptophytes, haptophytes and dinoflagellates, whereas it is Lhcq in ochrophytes[1,2,7,8,11–18] (Supplementary Fig. 11).

These cases illustrate how structural comparisons of composition, conformations and interactions can complement and shed light on phylogenetic hypotheses. Any accepted evolutionary model will need to be consistent with the protein and chromophore features revealed by high-resolution structures of PSI, PSII and other multi-subunit protein assemblies[1,2,7,8,11–18]. Structures from additional red-lineage clades, coupled with a better understanding of the evolutionary history of the specific FCPs found in the antennae and of the inferred ancestral organisms that were engulfed, are needed to fully elucidate the relationships in the red lineage.

Supplementary Methods:

Identification of plastocyanin and cytochrome c_6 coding sequences

Coding sequences for Pc and cyt c_6 were identified in the *Macrocystis pyrifera* genome using tBLASTn searches against the PhycoCosm database (Macrocystis pyrifera CI_03 v1.0). For Pc, the *Synechocystis sp.* PCC 6803 sequence (UniProt: P21697) was used as query, while for cyt c_6 the *Ectocarpus siliculosus* sequence (UniProt: A0A6G6D7E1) was employed. Searches were performed using with an expectation E-value 1.05×10^{-5} and the BLOSUM62 substitution matrix. We used the *M. pyrifera* "all-model-transcripts" dataset (release 202220914). Candidate coding sequences were translated into protein sequences, signal peptides were predicted, and mature protein sequences were extracted for downstream analysis.

Sequence alignment and electrostatic analysis of PsaF, cyt c_6, and Pc

PsaF sequences included *Synechocystis sp.* PCC 6803 (UniProt: P29256), *C. gracilis* (UniProt: A0A345U7L1), *G. sulphuraria* (UniProt: E3UIU5), and *Pisum sativum* (UniProt: E3UIU5). Cytochrome c_6 sequences were aligned from *M. pyrifera* (predicted, gene ID: DN39228 c0 g1 i3), *Phaeodactylum tricornutum* (UniProt: B5Y578) and *Synechocystis sp.* PCC 6803 (UniProt: P46445). Plastocyanin alignments included *M. pyrifera* (predicted, gene ID: DN119526 c0 g1 i1), *P. sativum* (UniProt: P16002), and *Synechocystis sp.* PCC 6803 (UniProt: P21697). All multiple sequence alignments were performed using ClustalW with standard parameters.

Structural modeling of electron donors and molecular docking

Pc and cyt c_6 predicted models were generated with AlphaFold3, including copper ion in Pc and heme C group in cyt c_6. Top-ranking models were selected per pLDDT confidence scores and stereochemical quality metrics from MolProbity. Conserved metal-binding motifs were confirmed by comparison to homologues (PDB: 1AG6 for Pc and 1CYI for cyt c_6). Electrostatic potential surfaces of PsaF, Pc and cyt c_6 were obtained using AMBER20 in ChimeraX. Docking simulations were performed between *M. pyrifera* PSI (this work) and predicted Pc and cyt c_6 models with HADDOCK 2.4, including rigid-body docking, semi-flexible refinement. Best-scoring clusters were selected per HADDOCK scores and interface consistency with known PSI-donor complexes from cyanobacteria and plants. Final docking poses were analyzed in ChimeraX to measure donor–P700 distances. Binding free energies were estimated using PISA.

Supplementary References:

1. Xu, C. *et al.* Structural basis for energy transfer in a huge diatom PSI-FCPI supercomplex. *Nat Commun* **11**, 5081 (2020).
2. Kato, K. *et al.* Structural basis for molecular assembly of fucoxanthin chlorophyll a/c-binding proteins in a diatom photosystem I supercomplex. *eLife* **13**, (2024).
3. Diesel, J. *et al.* A scaffolded and annotated reference genome of giant kelp (Macrocystis pyrifera). *BMC Genomics* **24**, 543 (2023).
4. Kühlbrandt, W., Wang, D. N. & Fujiyoshi, Y. Atomic model of plant light-harvesting complex by electron crystallography. *Nature* **367**, 614–621 (1994).
5. Iwai, M., Patel-Tupper, D. & Niyogi, K. K. Structural Diversity in Eukaryotic Photosynthetic Light Harvesting. *Annu Rev Plant Biol* https://doi.org/10.1146/annurev-arplant-070623-015519 (2024) doi:10.1146/annurev-arplant-070623-015519.
6. Kumazawa, M. *et al.* Molecular phylogeny of fucoxanthin-chlorophyll a/c proteins from Chaetoceros gracilis and Lhcq/Lhcf diversity. *Physiol Plant* **174**, e13598 (2022).
7. Kato, K. *et al.* Structure of a photosystem I supercomplex from Galdieria sulphuraria close to an ancestral red alga. *Science Advances* **11**, eadv7488 (2025).
8. You, X. *et al.* In situ structure of the red algal phycobilisome–PSII–PSI–LHC megacomplex. *Nature* **616**, 199–206 (2023).
9. Pi, X. *et al.* Unique organization of photosystem I-light-harvesting supercomplex revealed by cryo-EM from a red alga. *Proc Natl Acad Sci U S A* **115**, 4423–4428 (2018).
10. Antoshvili, M., Caspy, I., Hippler, M. & Nelson, N. Structure and function of photosystem I in Cyanidioschyzon merolae. *Photosynth Res* **139**, 499–508 (2019).
11. Zhao, L.-S. *et al.* Structural basis and evolution of the photosystem I–light-harvesting supercomplex of cryptophyte algae. *The Plant Cell* **35**, 2449–2463 (2023).
12. Zhang, S. *et al.* Growth phase-dependent reorganization of cryptophyte photosystem I antennae. *Commun Biol* **7**, 1–10 (2024).
13. He, F.-Y. *et al.* Structural insights into the assembly and energy transfer of haptophyte photosystem I–light-harvesting supercomplex. *Proceedings of the National Academy of Sciences* **121**, e2413678121 (2024).
14. Shen, L. *et al.* Structure and function of a huge photosystem I–fucoxanthin chlorophyll supercomplex from a coccolithophore. *Science* **389**, eadv2132 (2025).
15. Li, X. *et al.* Structures and organizations of PSI–AcpPCI supercomplexes from red tidal and coral symbiotic photosynthetic dinoflagellates. *Proceedings of the National Academy of Sciences* **121**, e2315476121 (2024).
16. Zhao, L.-S. *et al.* Architecture of symbiotic dinoflagellate photosystem I–light-harvesting supercomplex in Symbiodinium. *Nat Commun* **15**, 2392 (2024).
17. Nagao, R. *et al.* Structural basis for assembly and function of a diatom photosystem I-light-harvesting supercomplex. *Nat Commun* **11**, 2481 (2020).
18. Feng, Y. *et al.* Structures of PSI–FCPI from Thalassiosira pseudonana grown under high light provide evidence for convergent evolution and light-adaptive strategies in diatom FCPIs. *Journal of Integrative Plant Biology* **67**, 949–966 (2025).
19. Slater, B., Kosmützky, D., Nisbet, R. E. R. & Howe, C. J. The Evolution of the Cytochrome *c* 6 Family of Photosynthetic Electron Transfer Proteins. *Genome Biology and Evolution* **13**, evab146 (2021).

20. Peers, G. & Price, N. M. Copper-containing plastocyanin used for electron transport by an oceanic diatom. *Nature* **441**, 341–344 (2006).
21. Caspy, I. *et al.* Structure of plant photosystem I-plastocyanin complex reveals strong hydrophobic interactions. *Biochemical Journal* **478**, 2371–2384 (2021).
22. Bernal-Bayard, P. *et al.* Interaction of photosystem I from Phaeodactylum tricornutum with plastocyanins as compared with its native cytochrome c6: Reunion with a lost donor. *Biochimica et Biophysica Acta (BBA) - Bioenergetics* **1847**, 1549–1559 (2015).
23. Blankenship, R. E. *Molecular Mechanisms of Photosynthesis*. (Wiley, 2021).
24. Caspy, I., Borovikova-Sheinker, A., Klaiman, D., Shkolnisky, Y. & Nelson, N. The structure of a triple complex of plant photosystem I with ferredoxin and plastocyanin. *Nat. Plants* **6**, 1300–1305 (2020).
25. Strassert, J. F. H., Irisarri, I., Williams, T. A. & Burki, F. A molecular timescale for eukaryote evolution with implications for the origin of red algal-derived plastids. *Nat Commun* **12**, 1879 (2021).
26. Pietluch, F., Mackiewicz, P., Ludwig, K. & Gagat, P. A New Model and Dating for the Evolution of Complex Plastids of Red Alga Origin. *Genome Biol Evol* **16**, evae192 (2024).
27. Stiller, J. W. *et al.* The evolution of photosynthesis in chromist algae through serial endosymbioses. *Nat Commun* **5**, 5764 (2014).
28. Bodył, A., Stiller, J. W. & Mackiewicz, P. Chromalveolate plastids: direct descent or multiple endosymbioses? *Trends Ecol Evol* **24**, 119–121; author reply 121-122 (2009).
29. Shin, D. W., Chen, T., Letts, J. A. Mapping the evolution of mitochondrial complex I through structural variation. FEBS Letters in press. *FEBS Letters* **in press**.
30. Douglas, S. E. & Penny, S. L. The Plastid Genome of the Cryptophyte Alga, Guillardia theta: Complete Sequence and Conserved Synteny Groups Confirm Its Common Ancestry with Red Algae. *J Mol Evol* **48**, 236–244 (1999).
31. Vanselow, C., Weber, A. P. M., Krause, K. & Fromme, P. Genetic analysis of the Photosystem I subunits from the red alga, *Galdieria sulphuraria*. *Biochimica et Biophysica Acta (BBA) - Bioenergetics* **1787**, 46–59 (2009).
32. Puerta, M. V. S., Bachvaroff, T. R. & Delwiche, C. F. The Complete Plastid Genome Sequence of the Haptophyte Emiliania huxleyi : a Comparison to Other Plastid Genomes. *DNA Res* **12**, 151–156 (2005).
33. Terpis, K. X. *et al.* Multiple plastid losses within photosynthetic stramenopiles revealed by comprehensive phylogenomics. *Current Biology* **35**, 483-499.e8 (2025).
34. Liu, H. W. *et al.* A distinct LHCI arrangement is recruited to photosystem I in Fe-starved green algae. *Proceedings of the National Academy of Sciences* **122**, e2500621122 (2025).
35. Li, K. *et al.* Structure and energy transfer of a far-red–absorbing euglenophyte PSI–LhcE–LhcbM supercomplex. *Nat Commun* https://doi.org/10.1038/s41467-026-70067-1 (2026) doi:10.1038/s41467-026-70067-1.
36. Huang, G. *et al.* The architecture and energy transfer pathways of PSI–LHCI–LHCII in the phototrophic flagellate Euglena gracilis. *Nat Commun* https://doi.org/10.1038/s41467-026-70183-y (2026) doi:10.1038/s41467-026-70183-y.
37. Kumazawa, M. & Ifuku, K. Unraveling the evolutionary trajectory of LHCI in red-lineage algae: Conservation, diversification, and neolocalization. *iScience* **27**, 110897 (2024).
