# Supplementary material for: Structure of giant kelp Photosystem I-FCP uncovers drivers of antenna evolution across the red lineage
Source: Nat Commun. 2026 May 21;17:4772. doi: 10.1038/s41467-026-73499-x (PMC13219633; doi:10.1038/s41467-026-73499-x)
Supplement: Supplementary file 2 — Description of Additional Supplementary Files [file 41467_2026_73499_MOESM2_ESM.pdf]

Description of Additional Supplementary Files

File name: Supplementary Data 1
Description: Sequences of FCP and related LHC proteins used in phylogenetic analysis. FCP sequences for *Macrocystis pyrifera*, *Ectocarpus siliculosus*, *Chaetoceros gracilis*, *Thalassiosira pseudonana* and green algal LHC proteins used to root the trees in Supplementary Figure 9.

File name: Supplementary Data 2
Description: Membrane thickness of FCP and core helices across the red lineage.
Data File 2. Membrane thickness of FCP and core helices across the red lineage. Measurements of the membrane span of the PsaA/B and FCP helices A-C for subfamilies Lhcr, Lhcq, Lhcq-like, Lhcf measured from *Macrocystis pyrifera* (PDB: 9YGV, this work), *Emiliania huxleyi* (PDB: 9JJ8) and *Galdieria sulphuraria* (PDB: (9KC5)[7] shown in Fig. 3. To determine the span of the helices, measurements were taken using the distance tool in Coot and values were scaled relative to the field of view of the membrane in Inkscape. To determine the averages for each FCP subfamily, at least 3 measurements were taken for each helix of the different FCP subfamilies; when possible, at least three subfamily members were measured.
