# Supplementary material for: Structure of giant kelp Photosystem I-FCP uncovers drivers of antenna evolution across the red lineage
Source: Nat Commun. 2026 May 21;17:4772. doi: 10.1038/s41467-026-73499-x (PMC13219633; doi:10.1038/s41467-026-73499-x)
Supplement: Supplementary file 5 — Reporting Summary [file 41467_2026_73499_MOESM5_ESM.pdf]

nature portfolio

Corresponding author(s): Maldonado, Maria

Last updated by author(s): Mar 31, 2026

Reporting Summary

Nature Portfolio wishes to improve the reproducibility of the work that we publish. This form provides structure for consistency and transparency in reporting. For further information on Nature Portfolio policies, see our Editorial Policies and the Editorial Policy Checklist.

Statistics

For all statistical analyses, confirm that the following items are present in the figure legend, table legend, main text, or Methods section.

n/a	Confirmed	
☐	☒	The exact sample size (*n*) for each experimental group/condition, given as a discrete number and unit of measurement
☒	☐	A statement on whether measurements were taken from distinct samples or whether the same sample was measured repeatedly
☒	☐	The statistical test(s) used AND whether they are one- or two-sided<br>*Only common tests should be described solely by name; describe more complex techniques in the Methods section.*
☒	☐	A description of all covariates tested
☒	☐	A description of any assumptions or corrections, such as tests of normality and adjustment for multiple comparisons
☐	☒	A full description of the statistical parameters including central tendency (e.g. means) or other basic estimates (e.g. regression coefficient) AND variation (e.g. standard deviation) or associated estimates of uncertainty (e.g. confidence intervals)
☒	☐	For null hypothesis testing, the test statistic (e.g. *F*, *t*, *r*) with confidence intervals, effect sizes, degrees of freedom and *P* value noted<br>*Give P values as exact values whenever suitable.*
☒	☐	For Bayesian analysis, information on the choice of priors and Markov chain Monte Carlo settings
☒	☐	For hierarchical and complex designs, identification of the appropriate level for tests and full reporting of outcomes
☒	☐	Estimates of effect sizes (e.g. Cohen's *d*, Pearson's *r*), indicating how they were calculated

Our web collection on statistics for biologists contains articles on many of the points above.

Software and code

Policy information about availability of computer code

Data collection	CryoEM data were collected using a 300kV microscope equipped with a Falcon 4i detector operated in electron counting (EC) mode at SLAC National Accelerator Laboratory, Stanford, CA, USA. Data acquisition was performed using EPU software. A total dose of 50 $e^{-}Å^{-2}$ was fractionated into 40 frames over an exposure time of 4.95 s, resulting in a dose rate of 1.25 $e^{-}Å^{-2}frame^{-1}$. Movies were collected at a physical pixel size of 1.217 Å, corresponding to a magnification yielding 1.27 Å $pixel^{-1}$ on the detector. A total of 15,690 micrographs were acquired, with a fluence of 6.82 $e^{-}pix^{-1}$ (10.10 $e^{-}Å^{-2}s^{-1}$).
Data analysis	Motion correction and contrast transfer function (CTF) estimation were performed using CTFFIND4.1 in Relion v4.0.1. Particle picking was initially guided by manual picking and subsequently used to train a Topaz model (73). Particles were transferred to cryoSPARC v4.4.1 for successive rounds of 2-dimensional classifications and ab-initio volume generation before heterogeneous refinement and re-extraction of a curated particle set at the original pixel size. Non-uniform refinement was performed, followed by core-focused Local Refinement using soft binary masks around different areas, generated in UCSF ChimeraX v1.10.1

For manuscripts utilizing custom algorithms or software that are central to the research but not yet described in published literature, software must be made available to editors and reviewers. We strongly encourage code deposition in a community repository (e.g. GitHub). See the Nature Portfolio guidelines for submitting code & software for further information.

Data

Policy information about availability of data

All manuscripts must include a data availability statement. This statement should provide the following information, where applicable:

- Accession codes, unique identifiers, or web links for publicly available datasets
- A description of any restrictions on data availability
- For clinical datasets or third party data, please ensure that the statement adheres to our policy

The atomic coordinates for the M. pyrifera PSI-FCP supercomplex have been deposited (PDB: 9YGV). Maps corresponding to the composite map (EMDB: 76146) as well as 19 focused-refinement maps for each component of the composite map have been deposited. Details are available in Supplementary table 2. The raw cryoEM data has been deposited (EMPIAR:12998).

Research involving human participants, their data, or biological material

Policy information about studies with human participants or human data. See also policy information about sex, gender (identity/presentation), and sexual orientation and race, ethnicity and racism.

Reporting on sex and gender	n.a.
Reporting on race, ethnicity, or other socially relevant groupings	n.a.
Population characteristics	n.a.
Recruitment	n.a.
Ethics oversight	n.a.

Note that full information on the approval of the study protocol must also be provided in the manuscript.

Field-specific reporting

Please select the one below that is the best fit for your research. If you are not sure, read the appropriate sections before making your selection.

☒ Life sciences ☐ Behavioural & social sciences ☐ Ecological, evolutionary & environmental sciences

For a reference copy of the document with all sections, see nature.com/documents/nr-reporting-summary-flat.pdf

Life sciences study design

All studies must disclose on these points even when the disclosure is negative.

Sample size	No sample-size calculations were performed, sample size was chosen based on the practical considerations of sample availability and experimental design. Each chloroplast isolation preparation beings from 1 kg of kelp material, which corresponds to multiple blades across multiple organisms.
Data exclusions	No statistical data exclusion.
Replication	Chloroplast isolations, PSI detergent extractions and purifications were reproducibly performed for >10 instances until 3 adequate cryoEM grids were obtained in reproducible sample preparation conditions.
Randomization	Only one experimental group. Multiple blades originating from multiple kelp organisms were used for each chloroplast isolation preparation.
Blinding	Investigators were not blinded to the sample allocations because all the samples were considered equivalent and conditions were predetermined and analysed identically.

Reporting for specific materials, systems and methods

We require information from authors about some types of materials, experimental systems and methods used in many studies. Here, indicate whether each material, system or method listed is relevant to your study. If you are not sure if a list item applies to your research, read the appropriate section before selecting a response.

Materials & experimental systems

n/a	Involved in the study
☒	☐ Antibodies
☒	☐ Eukaryotic cell lines
☒	☐ Palaeontology and archaeology
☒	☐ Animals and other organisms
☒	☐ Clinical data
☒	☐ Dual use research of concern
☐	☒ Plants

Methods

n/a	Involved in the study
☒	☐ ChIP-seq
☒	☐ Flow cytometry
☒	☐ MRI-based neuroimaging

Dual use research of concern

Policy information about dual use research of concern

Hazards

Could the accidental, deliberate or reckless misuse of agents or technologies generated in the work, or the application of information presented in the manuscript, pose a threat to:

No	Yes
☒	☐ Public health
☒	☐ National security
☒	☐ Crops and/or livestock
☒	☐ Ecosystems
☒	☐ Any other significant area

Experiments of concern

Does the work involve any of these experiments of concern:

No	Yes
☒	☐ Demonstrate how to render a vaccine ineffective
☒	☐ Confer resistance to therapeutically useful antibiotics or antiviral agents
☒	☐ Enhance the virulence of a pathogen or render a nonpathogen virulent
☒	☐ Increase transmissibility of a pathogen
☒	☐ Alter the host range of a pathogen
☒	☐ Enable evasion of diagnostic/detection modalities
☒	☐ Enable the weaponization of a biological agent or toxin
☒	☐ Any other potentially harmful combination of experiments and agents

Plants

Seed stocks	n.a. (We recognise that brown algae are not plants, but the plant category seemed more appropriate than "animals and other organisms").
Novel plant genotypes	n.a.
Authentication	n.a.<br>Macrocystis pyrifera samples are obtained from Monterey Bay Seaweeds, a commercial enterprise run by expert phycologist Dr Michael Graham. The enterprise is approved for sales for human consumption. Moreover, the cryoEM density maps allow the determination of protein sequences of the subunits of the complex, which matched the expected sequence found in the M. pyrifera genome.
